# Kartograf: A Geometrically Accurate Atom Mapper for Hybrid-Topology Relative Free Energy Calculations

**DOI:** 10.1021/acs.jctc.3c01206

**Published:** 2024-02-08

**Authors:** Benjamin Ries, Irfan Alibay, David W. H. Swenson, Hannah M. Baumann, Michael M. Henry, James R. B. Eastwood, Richard J. Gowers

**Affiliations:** † Medicinal Chemistry, Boehringer Ingelheim Pharma GmbH & Co KG, Birkendorfer Str 65, 88397 Biberach an der Riss, Germany; ‡ Open Free Energy, Open Molecular Software Foundation, Davis, 95616 California, United States; § Computational and Systems Biology Program, Sloan Kettering Institute, Memorial Sloan Kettering Cancer Center, New York, 1275 New York, United States

## Abstract

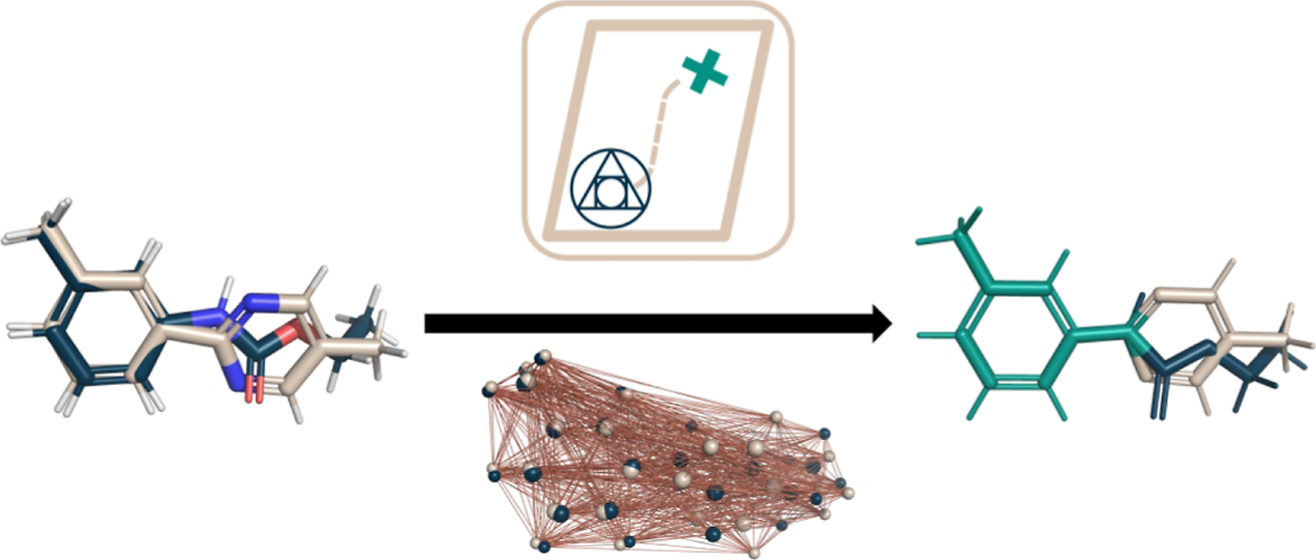

Relative binding free energy (RBFE) calculations have emerged as a powerful tool that supports ligand optimization in drug discovery. Despite many successes, the use of RBFEs can often be limited by automation problems, in particular, the setup of such calculations. Atom mapping algorithms are an essential component in setting up automatic large-scale hybrid-topology RBFE calculation campaigns. Traditional algorithms typically employ a 2D subgraph isomorphism solver (SIS) in order to estimate the maximum common substructure. SIS-based approaches can be limited by time-intensive operations and issues with capturing geometry-linked chemical properties, potentially leading to suboptimal solutions. To overcome these limitations, we have developed Kartograf, a geometric-graph-based algorithm that uses primarily the 3D coordinates of atoms to find a mapping between two ligands. In free energy approaches, the ligand conformations are usually derived from docking or other previous modeling approaches, giving the coordinates a certain importance. By considering the spatial relationships between atoms related to the molecule coordinates, our algorithm bypasses the computationally complex subgraph matching of SIS-based approaches and reduces the problem to a much simpler bipartite graph matching problem. Moreover, Kartograf effectively circumvents typical mapping issues induced by molecule symmetry and stereoisomerism, making it a more robust approach for atom mapping from a geometric perspective. To validate our method, we calculated mappings with our novel approach using a diverse set of small molecules and used the mappings in relative hydration and binding free energy calculations. The comparison with two SIS-based algorithms showed that Kartograf offers a fast alternative approach. The code for Kartograf is freely available on GitHub (https://github.com/OpenFreeEnergy/kartograf). While developed for the OpenFE ecosystem, Kartograf can also be utilized as a standalone Python package.

## Introduction

Drug design is a complex process that necessitates balancing cost, speed, and accuracy to achieve efficiency.^1,2^ In recent years, in silico methods have become increasingly important in enhancing these factors.^1,3−7^ A fundamental property in the multifaceted drug design process is potency, alongside ADME and synthetic accessibility.^2,8−11^ To evaluate the potency of potential drug candidates in silico, free energy calculation methods, including simulations on the theoretical level of molecular mechanics or quantum mechanics, are currently the state-of-the-art that promise the most accurate estimates.^7,12^ Furthermore, large efforts are undertaken to automatize such approaches.^13−22^ In order to rank the most promising drug candidates by potency with in silico methods, ligands are typically ranked based on their calculated binding free energies.^3,23^

**Figure 1:**
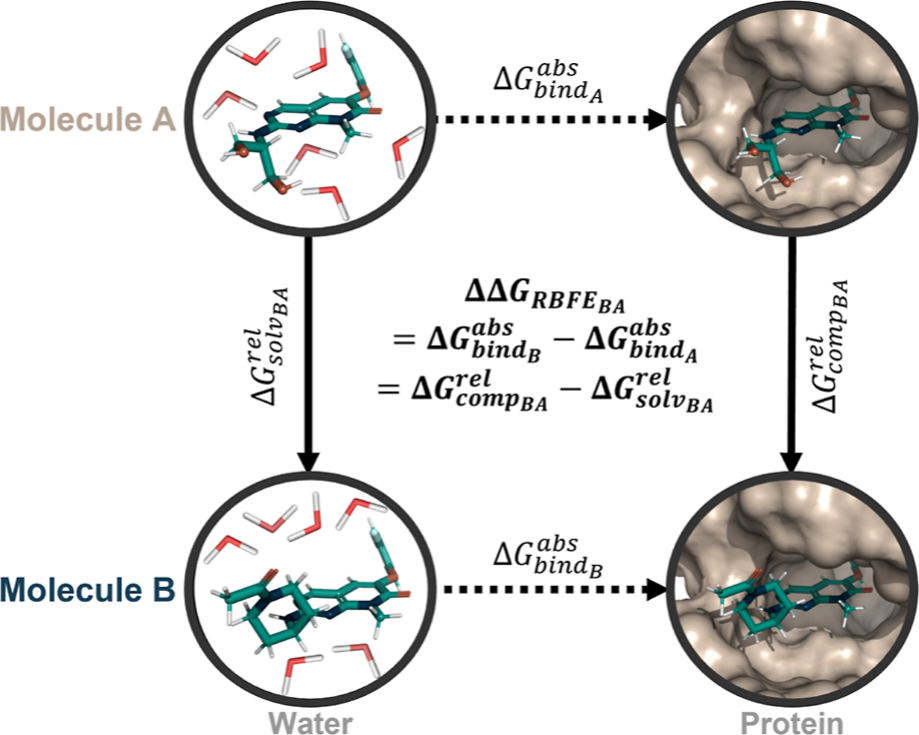
Binding free energy thermodynamic cycle can be employed to calculate RBFEs (ΔΔ*G*_RBFE_**BA**__) by comparing two molecules **A** and **B**. The absolute free energy differences  and  represent the change of molecules **A** and **B**’s environment from water to the protein complex and can be combined to calculate . Alternatively, calculating the RBFE between **A** and **B** in a specific environment  can often be used to more efficiently calculate . The environments, such as water and the complex, can be exchanged with, e.g., the combination of vacuum and water in order to retrieve RHFEs.

Binding free energy calculations can be categorized into two types depending on the calculated property: relative binding free energies (RBFEs) or absolute binding free energies (ABFEs).^12^ ABFEs provide direct insight into the binding free energy Δ*G*_**A**_^bind^ of ligand **A** but are computationally expensive as the entire ligand perturbation is simulated from a bound to an unbound state by turning all interactions of the ligand off and on again.^3,24−27^ In contrast, RBFEs leverage alchemical transformations of the ligands, representing a nonphysical transformation of a system between ligand **A** and ligand **B** (end-states), enabling fewer atomic mutations and leading to a smaller computational effort.^3^ The results describe the alchemical changes in water ΔΔ*G*_**BA**_^solv^ and in the complex ΔΔ*G*_**BA**_^complex^ with the target protein, which can be used to calculate the binding free energy difference between the two ligands, ΔΔ*G*_**BA**_^bind^, required to rank the potential drug candidates (see Figure 1).^12^

A crucial component of an RBFE calculation is the system representation.^28^ In previous work, Ries et al. identified three distinct ways of representing the system from literature: single topology,^29−31^ hybrid topology,^32,33^ and dual topology.^28,30,34^

**Figure 2:**
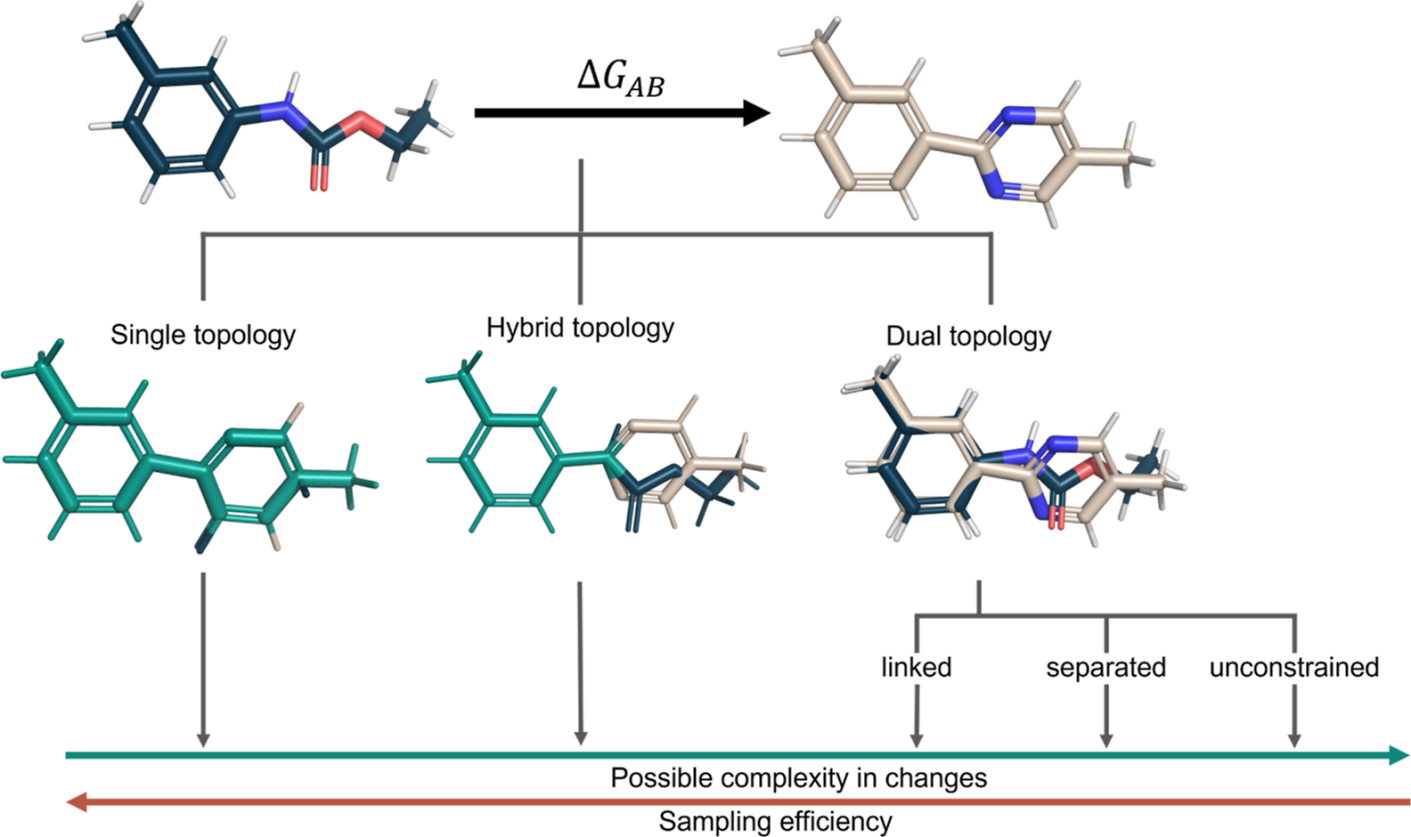
When considering end-state **A** (depicted in dark blue) and end-state **B** (depicted in beige), there exist three distinct approaches for representing the systems in free energy calculations: single topology, employing a minimal set of coordinates for both end-states (green merged atoms); hybrid topology, which merges shared parts into a core region and uses dual-topology-like regions for differing regions; and dual topology, where the coordinates of both end-states are kept separate. In the realm of dual topology, various approaches are found in literature, including the unconstrained case, the separated dual-topology approach using orientational restraints, and the linked dual-topology approach, connecting the end-states with restraints.

Dual-topology variants, such as linked dual topology and separated dual topology, represent the changing molecules in the end-states by independent sets of coordinates, allowing a straightforward representation of large ligand transformations with the respective restraint placement algorithm.^22,35−40^ The opposite extreme to dual topology is the single-topology approach, where a minimal set of coordinates represents both end-states. The single-topology approach reduces the separation of the phase space between the two end-states significantly and is proposed to give an efficiency win compared to dual-topology approaches but allows for less diverse molecule changes.^30,31,41,42^ The hybrid-topology approach offers an improved calculation efficiency compared to that of dual-topology approaches as the coordinates of shared parts of the molecules are merged to the so-called core region, and differing parts of the molecule are represented by dual-topology-like regions.^32,33,43−48^ This approach is an intermediate between dual- and single-topology approaches. Its primary challenge is to find the mapping of atoms for the core region (see Figure 2).^32^

Many atom mapping algorithms for free energy calculations use cheminformatics-driven approaches focusing on 2D chemical properties and SIS searches in order to estimate the maximum common substructure (MCS) serving as the core region, as seen in Lomap,^32^ pmx,^49^ fkckombu,^50^ FESetup,^51^ ProtoCaller,^52^ or SMArt.^53−55^ Alternatively, atom mapping approaches could be focused on the 3D geometry of the system.^56^ One benefit of such a method is the potential to bypass expensive SISs, which are trying to solve an NP-complete problem.^57^

Some atom mapping approaches based on SIS, such as pmx and Lomap, actually combine the 2D cheminformatics and the 3D geometry aspects but focus on the SIS algorithm.^32,49^

From practical considerations, the optimal atom mapper algorithm for a given application might depend on the intention of the modeler. For instance, if a given set of coordinates needs to be preserved as much as possible because they represent a binding mode or a special design idea, then a 3D geometry-focused approach might be the preferred method. However, if an optimal overlap of the molecules and their chemical properties is important, cheminformatics-based approaches may be more suitable.^56^

In this work, we present Kartograf, a package that implements an optimal atom mapping algorithm based on 3D geometric information. The proposed algorithm essentially addresses a bipartite graph matching problem using the Jonker–Volgenant algorithm, which has a computational worst-case complexity of *O*(*n*) = *N*^3^.^58,59^ We showcase features of our approach through theoretical examples illustrating its potential advantages. Furthermore, we will validate our atom mapper with test data sets for relative hydration and binding free energies. Kartograf is seamlessly integrated into the OpenFE environment but can alternatively be installed as a standalone package via PyPi or the conda-forge.^60,61^ The source code is readily available under the MIT license on GitHub at https://github.com/OpenFreeEnergy/kartograf.

## Theory

### Perturbations in Free Energy Calculations

The primary challenge in establishing RBFE calculations using a hybrid-topology approach is identifying the largest shared core region in both end-states. The complexity of the phase space can be reduced by merging the atom coordinates of the found core region and therefore enhance the sampling efficiency.^30,31,33,62^ Optimal merging of coordinates aims to minimize atomic perturbations and does not reduce the phase space overlap between end-states, therefore lowering the sampling cost of retrieving converged free energy estimations.^27^ The following objectives can be outlined for ligand atom mapping.

### Minimizing the Number of Dummy Atoms

The noninteracting dummy atoms are used to make transformations possible between molecules that do not share the same number of atoms or do not overlap well in phase space.^31,41^ Atoms that transition from a dummy state to a physical state may need to displace surrounding atoms, such as solvent or protein components, in order to generate the necessary volume. Nonbonded interactions, such as van der Waals and electrostatic interactions, play a critical role in creating the necessary cavity.^27,62^ However, the more dummy atoms present in a state, the larger the volume or cavity that needs to be generated, making the transformation more challenging.^27,62^ Incorporating soft-core potentials into the calculations can help avoid singularities.^63,64^

### Avoiding Flexibility Changes in Mapped Regions

If the flexibility of a mapped region varies between end-states, simulations are likely to lead to convergence issues. The difference in flexibility can be induced by bond-order changes, ring-breaking, or even ring-size changes. Distinct phase spaces may be explored by the different end-states, and coupling them through atom mapping might bias the states into nonoptimal phase space regions, resulting in poor representation of the end-states.^65^

### Minimizing the Spatial Distance between Atoms Being Mapped

The final challenge identified pertains to the mapping of atoms that have large spatial distances from each other. This is particularly problematic when the input coordinates describe binding modes or specific desired 3D contexts. For small atom-pair distances, the perturbation might be minimal and acceptable. However, for larger distances, a modified binding mode or an incorrect stereochemistry might be sampled. This can be a significant issue in protein pockets, where the environment typically adapts slowly to ligands and steric hindrance limits binding mode sampling.

Additionally, some mapping algorithms might introduce coordinate shifts or further alignments to the molecules being mapped, which can potentially lead to clashes with the environment, further increasing the system perturbation.^32^ To mitigate this, SIS-based atom mappers like Lomap and PMX have introduced distance cutoffs to reduce the atom displacement caused by mapping.^32,46^ Still, stereochemistry remains a challenge for these SIS-based mappers.

It is important to note that all three goals, minimizing the number of dummy atoms, avoiding flexibility changes in mapped regions, and minimizing the spatial distance between atoms being mapped, can partially conflict with each other. The final mapping will need to find a balance among all the rules to be feasible.

### Kartograf’s Atom Mapping Algorithm

Kartograf’s atom mapping algorithm is based on minimizing the spatial distance between the mapped atoms to identify the MCS (see section). The initial assumption of the atom mapper is that the coordinate space of the end-state molecules has a high spatial overlap and is considered to be static for the mapping process.

This high overlap can be assumed because the input to the free energy pipelines typically comes from, for example, docking procedures into the same coordinate space, a previous ligand alignment, or manual modeling.

The atom mapping approach of Kartograf does not take into account atom types and atom properties in the atom mapping itself. This challenge is addressed by network generation and the mapping scoring method. These methods are the only ways to avoid unfavorable atom type changes, assuming that the positions of the molecules are fixed.

### Procedure

#### Solving the Bipartite Graph Matching Problem for Atom Mappings

**Figure 3:**
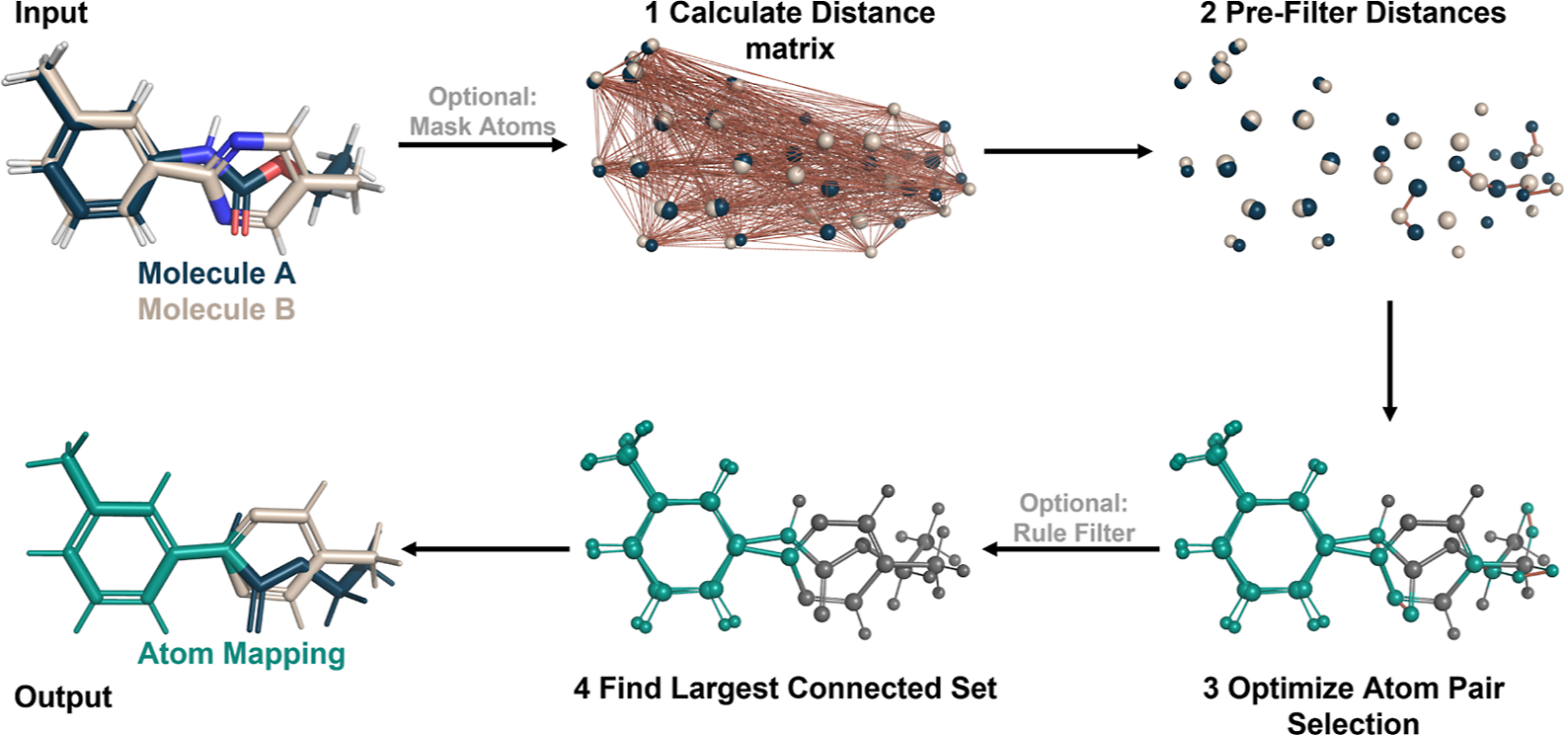
Kartograf’s atom mapping approach assumes as input a well-aligned pair of molecules **A** (dark blue) and **B** (beige). The approach calculates distances (red) between all atoms in both ligands. Subsequently, a distance prefilter is applied, significantly reducing the search scope by allowing only reasonable atom mapping distances (default cutoff: 0.95 Å). Optionally, users can mask atoms to narrow down the search space. An initial optimal mapping (green) is determined based on distances using the Kuhn–Munkres algorithm.^66−68^ Additionally, users can apply filter rules to the initial mapping, such as restricting hydrogen-to-heavy atom mapping and preventing ring breaks. From the resulting mapping, the largest connected set is returned as the mapped core region of the hybrid-topology approach.

As input, the atom mapper uses two molecule conformations. First, the two conformations **A** and **B** are translated into independent sets of nodes **N**_**A**_ and **N**_**B**_ based on the coordinates indicating the atom positions of the molecules. Next, a complete weighted bipartite graph is calculated by generating a full distance matrix from each node in **N**_**A**_ to each node in **N**_**B**_ (Figure 3 step 1). This step is followed by a filtering step that removes edges that have a distance weight longer than a given threshold (default: 0.95 Å), resulting in a sparser bipartite graph (Figure 3, step 2).

The final perfect matching problem is solved with the Kuhn–Munkres algorithm^66−68^ for optimal linear sum assignments. The algorithm reduces the sparse bipartite graph edges to a perfect matching of this graph, where each node of the graph has a degree of one (Figure 3-3). The gained atom pair set **p**_**AB**_ can be further reduced by rules-based filters (i.e., by not allowing ring-breaking/ring-size changes in the atom-pair sets; more below). We consider this algorithmic approach to be an exact maximum common edge subgraph approach if and only if the atom alignment is ideal for both molecules as it returns the optimal matching for a given set or coordinates.^54^

The previously mentioned challenges of stereochemistry and molecule symmetries in atom mappings are intrinsically dealt with in this initial mapping due to the geometric matching approach as long as the atom alignment is optimal and the distance threshold is smaller than the smallest covalent bond.

#### Finding the Hybrid-Topology Core Region

The perfect matching, denoted as **p**_**AB**_, serves as an optimal starting point for identifying the core region of the hybrid topology. A key requirement for this core region is that it should only consist of atoms connected by covalent bonds in both **A** (**a**_**A**i_^connected^) and **B** (**a**_**B**j_^connected^) to avoid the resonance effects of disjunctive mapping regions during the simulation. The perfect matching obtained in the previous step may result in several disconnected sets of atoms for both **A** and **B** in **p**_**AB**_ (see Figure 3, step 3, which yields four connected sets for **A** and three in **B**). In the final step, the largest overlap of two **a**_**A**i_^connected^ and **a**_**B**j_^connected^ in **p**_**AB**_ is found. The overlap is measured in the number of edges the two sets can form together in **p**_**AB**_. The identified sets with the largest connected mapping region are then chosen as the core region for the hybrid-topology approach (see Figure 3, step 4).

#### Additional Filters by Chemical Rules/Premapping

In an effort to optimize atom mappings concerning the previously mentioned objectives, we offer users the ability to filter the resulting atom mappings using rule-based filters (see Figure 3, steps 3 and 4). Several filters have already been implemented, such as those that prevent element changes in the mapping, hydrogen atoms being mapped onto heavy atoms, and alterations in molecule flexibility, for example, by ring-breaking or molecule ring-size changes. The latter can cause issues in many MD codes as bonded terms are typically left unscaled for efficiency reasons, leading to singularities during the simulations. The benefits of avoiding ring breaks have been described in the literature by Liu et al.^65^ One solution to this problem was introduced by Wang et al., who proposed softbond-bonded potentials that enable scaffold hops in core regions.^69^ Alternatively, such mappings can simply be avoided if a hybrid-topology core region remains.

Furthermore, users have the option to provide premappings, allowing for the iterative generation of the mappings or ensuring the mapping of specific substructures (see Figure 3, steps 1 and 2). A more detailed explanation of the practical usage is given in the documentation of Kartograf (https://kartograf.readthedocs.io/en/latest/tutorial/custom_filters.html).

### Mapping Scoring Metrics Taking Geometry into Account

The quantitative evaluation of a given mapping remains a critical question in free energy calculations beyond the mapping phase. The number of potential mappings for a set of *N* ligands is *N*(*N* – 1)/2.^32^ However, to generate a comprehensive ligand ranking, only a set of *N* – 1 mappings is necessary. An efficient network with *N* – 1 mappings can be constructed with the minimal spanning tree (MST) approach, which ideally uses the best *N* – 1 mappings of all possible mappings. Additional edges are often incorporated into perturbation networks to enhance the robustness of the free energy estimates. This strategy is evident in cycle closure approaches, for instance.^69−71^

To automatically obtain a quality assessment of mappings, various scoring approaches have been proposed in the past, such as Lomap and machine-learning approaches by Scheen et al.^32,70^ In this study, we investigate geometry-focused metrics to analyze the mappings, which could be of potential interest to the development of mapping scoring functions, like the mapping-based root-mean-square deviation (RMSD), the mapped volume ratio (MVR), the mapped atom ratio (MAR), the shape mismatch score (SMS), and the shape overlap score (SOS).

**Figure 4:**
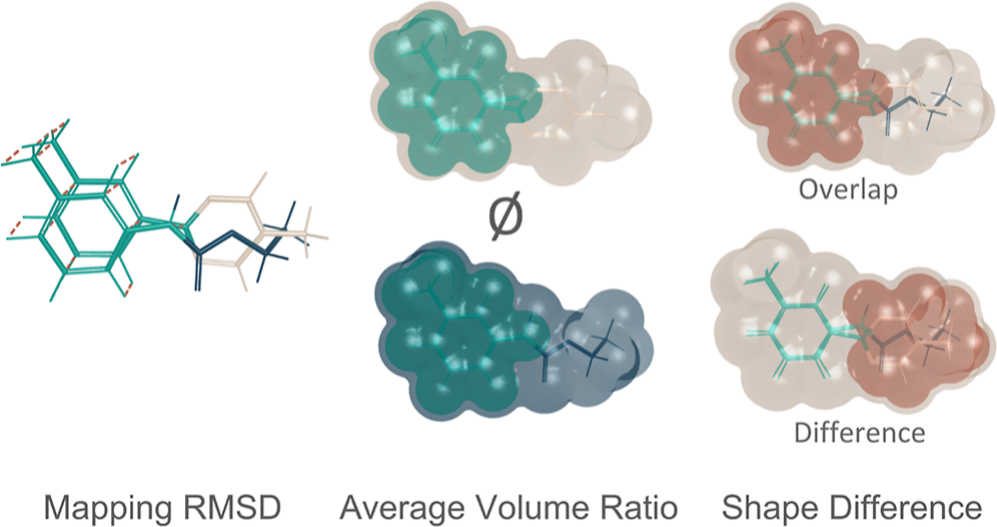
Geometry-based scores are used to assess the mappings. The mapping RMSD describes the atom displacement due to the mapping from one state to the other. The average volume ratio quantifies the extent to which the mapped region’s volume overlaps with the entire molecules, averaged across both molecules. Lastly, the shape difference is calculated for the mappings, comparing the shape difference of the mapped region and the whole molecules. Two distinct approaches were tested with varying emphases: one centered on overlap and the other emphasizing differences of the molecule shapes.

The mapping-based RMSD calculates the distance atoms need to travel from their original position to the resulting mapped position (refer to Figure 4, mapping RMSD). This score seeks to penalize large atomic displacements that may require excess configurational sampling.

MVR quantifies the shape overlap of the mapped region and the two molecules on average. In this case, a larger ratio is preferred as it indicates a larger overlap of the two ligands in the mapping. For planar molecules, this score is expected to highly correlate with the MAR or the maximum common substructure rule from the Lomap scorer. However, if a molecule with a fold or significant 3D structure is involved, it might diverge from the simpler metric (refer to Figure 4, average volume ratio).

The final metric added is shape-based and illustrates the difference between the mapped region and the original molecules (refer to Figure 4, difference). Here, we evaluate two different approaches to this score from the package rdkit:^72^ in the first mode, the focus is on molecule shape differences called SMS using the shape protrude distance difference of the molecules, and in the second mode, the focus is on molecule shape overlap called SOS by calculating the Tanimoto shape distance of the two molecules.

### Comparing Mappings

To quantitatively compare mappings generated by different methods, correlations of the number of mapped atoms and the Jaccard similarity coefficient (JCS) were used. The JCS indicates the diversity of mapped atom pairs of mapping *A* and mapping *B* (see eq 1). A score of 1 translates to two identical mappings, and a score of 0 translates to two completely different mappings.
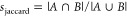
1

## Methods

### Implementation

Kartograf was written with Python 3^73,74^ in an object-oriented style. The source code and documentation are available on GitHub (https://github.com/OpenFreeEnergy/kartograf). The package can be used either as a standalone package with PyPi^60^ or the conda-forge project channel^61^ or from the OpenFE environment (https://github.com/OpenFreeEnergy/openfe).^75^ The Kartograf package uses NumPy^76^ for vector calculations and SciPy^59^ for the Kuhn–Munkres algorithm and convex hull volume determination^77^ in the MVR. The RDKit^72^ is heavily used for the representation of molecules and the score calculations, like the shape-difference scores or RMSD. The basic class types used in Kartograf were derived from the Grand Unified Free Energy (gufe) package, which provides standardized base types for classes in the OpenFE environment (https://github.com/OpenFreeEnergy/gufe).

### Mapping Approaches

In this study, the performance of Kartograf’s atom mapper was tested along with two alternative approaches representing different algorithmic flavors: a 2D SIS-only variant of the Lomap atom mapper (2D Lomap) and a mixed variant using both SIS and geometry aspects (default for Lomap-3D Lomap here) to estimate the MCS. The Lomap settings were used as defined in the Lomap Github repository (https://github.com/OpenFreeEnergy/Lomap), for the Lomap-3D approach the *threed* option was activated.

### Simulation Details

The following simulations were all conducted with the OpenFE release 0.10.1^75^ and the contained OpenFE CLI tools directly enabling the usage of the OpenFE Relative Free Energy (RFE) protocol.^78^ OpenFE was used for the simulations and preparations of OpenMM 8.0.0^79^ using the GPU code, OpenMMTools,^80^ and the OpenFF Toolkit 0.13.0.^81^ We note that the OpenFE RFE protocol is based on the Perses toolkit.^78^

The systems, except the vacuum systems, were first solvated using TIP3P waters^82^ up to a distance of 1.2 nm from the solute, directly defining the cubic periodic simulation box. Afterward, the systems were neutralized and set to an ion concentration of approximately 0.15 M with sodium and chloride ions.

For the ligand parameterization, OpenFF 2.1.0^83^ was used, and for the protein, AMBERFF14SB.^84^ To allow an integration speedup, the masses of the hydrogens were set to 3.0 amu following the hydrogen mass repartitioning (HMR) scheme.^85,86^ During any system simulation or optimization of solvated systems, the short-range nonbonded cutoff was set to 1 nm with long-range interactions handled using the PME scheme.^87^ For vacuum simulations in the relative hydration free energies (RHFE) calculations, no nonbonded cutoff was applied. All simulations employed constraints for hydrogen-containing bonds using SHAKE for solutes and SETTLE for water molecules. The tolerance was set to 10^–6^ kJ mol^–1^ nm^–1^ for the SHAKE^88^ and SETTLE^89^ combination used by OpenMM.

After the system setup, an energy minimization with an implementation of the L-BFGS Optimizer^90^ was performed for a maximum of 5000 steps. Following this, the system was replicated for 11 λ that forms the transformation path between end-state A and end-state B. The transformation is described by a linear coupling between both end-states with the extreme points of 0 and 1.^91^ To avoid singularities during the transformations, LJ softcore interactions as defined by Gapsys et al. were applied for the Lennard-Jones nonbonded terms with an α of 0.85 and a σ of 1.0.^64,92^

Each replicate was equilibrated with the corresponding λ-value for 1 ns. For the equilibration and the final production run, a Langevin integrator^93^ was used with an integration step of 4 fs due to the HMR scheme and a collision frequency of 1.0 ps^–1^. The temperature of the system was intrinsically kept at 298.15 K by the Langevin integrator. The pressure was kept at 1 bar with a Monte Carlo Barostat^94,95^ that was coupled every 1 ps. Additionally, a λ-dependent Hamiltonian replica exchange^96,97^ scheme was applied. The number of replicas equaled the number of λ-points, and after each 1 ps, a Metropolis–Hastings–Monte Carlo move tried to perform an all-to-all exchange scheme on the replicas.^98,99^ The production runs were performed to generate 5 ns simulations.

Finally, the free energies of each transformation were estimated using MBAR^100^ via pymbar.^101^

The whole procedure was repeated three times with different random seeds to assess the sampling uncertainty of the approach. Cinnabar^102^ was used for the network-wide free energy analysis over a full set of ligands. The bootstrap error estimate for the statistics (such as the root-mean-square error (RMSE), mean unsigned error (MUE), Pearson correlation coefficient (ρ_Pears_), and Kendall’s tau (τ_Kendall_)) was estimated over all replicates with SciPy.^59^

## Test Systems

### RHFE System

**Figure 5:**
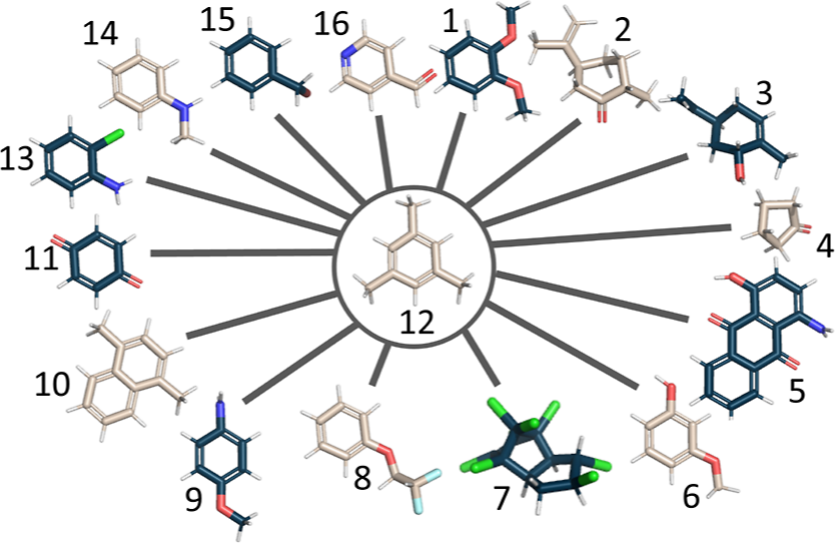
RHFE data set molecules represent a complex set of transformations that include R-group modifications, ring-system expansions, and ring-size changes. The shown radial network layout was used by Ries et al.,^28^ but ligands 7 and 4 were excluded from the data set as the current implementation of the RFE protocol in OpenFE does not allow ring-breaking. Adapted with permission from Ries et al. Copyright 2022 Springer Nature.^28^

In order to test the robustness and the outcome of the mapping approach, the RHFEs of the benzene set of Ries et al. were calculated.^22,28,91,103,104^ In the benzene RHFE data set, a large variety of transformation types occur, including R-Group changes, ring system growth, and ring hybridization changes (Figure 5).

Two different types of free energy perturbation networks were investigated: the original radial network by Ries et al. and MST networks generated using both the different mapping approaches and the Lomap scoring function. From the networks, ligands 7 and 4 were excluded as the current OpenFE RFE protocol implementation did not allow ring-breaking. The molecule coordinates were used from the work of Ries et al.^28^

### Protein Complex Systems

**Figure 6:**
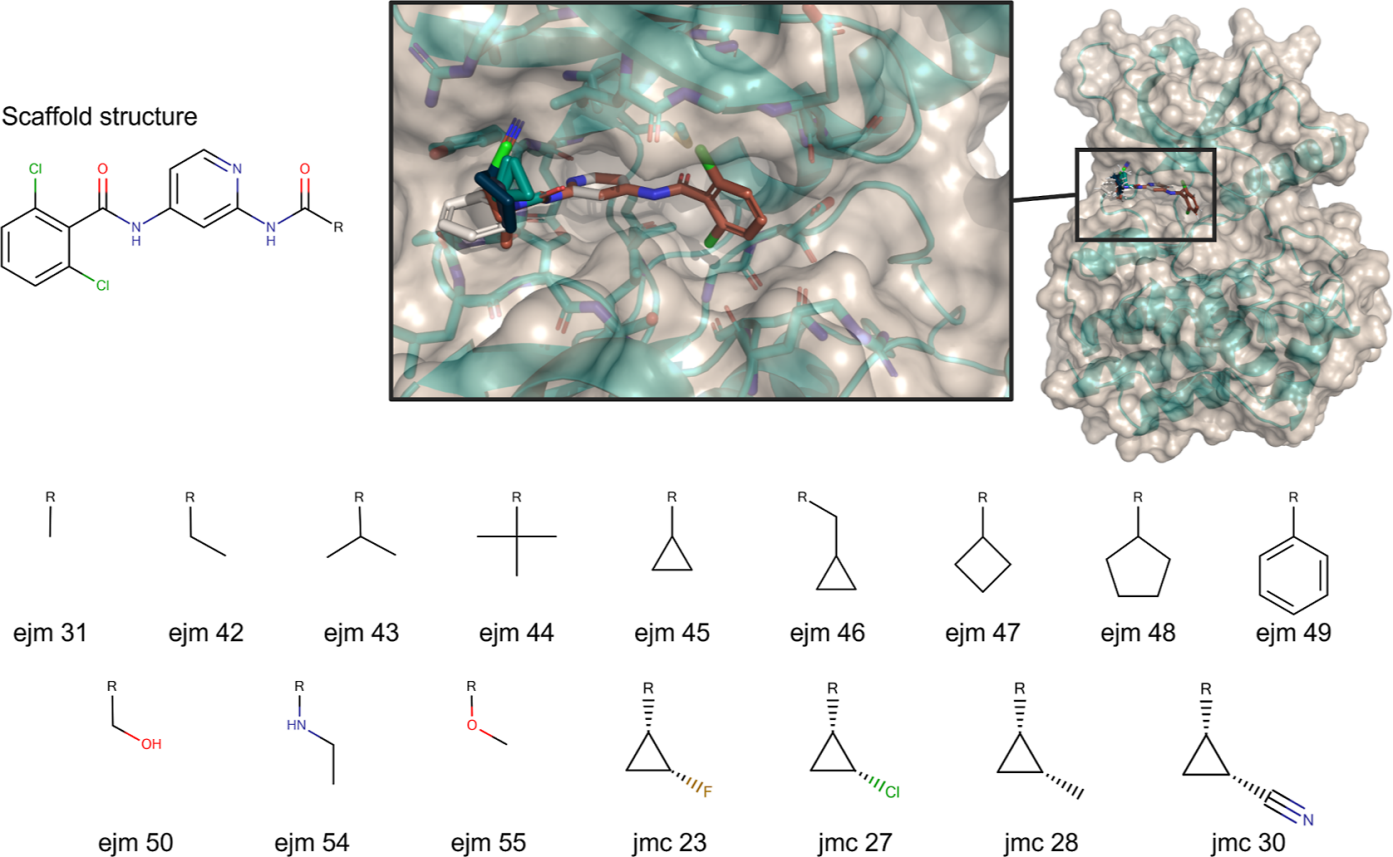
TYK2 test set is a small benchmarking test set, containing R-group changes in one location with a large common scaffold. The starting structures for the complex were taken from Hahn and Wagner.^105^

The tyrosine kinase 2 (TYK2) and hypoxia-inducible factor 2 alpha (HIF2A) test set coordinates and experimental data were derived from the protein–ligand benchmark (PLB).^69,105−109^ The systems cover ring-breaking, R-group changes, and ring-size changes. In the case of TYK2, all molecule changes are localized in the same molecule region and all atoms have a very high overlap, making the system a very well-behaved system (Figure 6). In contrast, HIF2A spreads the molecule transformations over the whole molecule, leading to much more difficult transformations (Figure S1).

The protein complex and the RHFE systems can be retrieved from the openfe-benchmarks release v.0.1.0 (https://github.com/OpenFreeEnergy/openfe-benchmarks).

### Protein Mutation Mapping

As a potential outlook for an expanded use-case of Kartograf, the protein mapping mutation test system was added. In the protein mutation system, aspartate 153 of TYK2 was mutated to a tyrosine (Figure S2). Note that no free energy calculation was performed with the protein mutation test system.

## Results and Discussion

### Assessment of Mapping Generated by the Mapping Approaches

To evaluate the performance of each mapping approach, we applied them to each possible ligand pair of the four different test systems. All mappers were able to find mappings for the provided data sets, except for the protein single residue mutation system, in which only Kartograf was able to converge on a mapping.

### Comparing Mappings of Different Approaches

**Figure 7:**
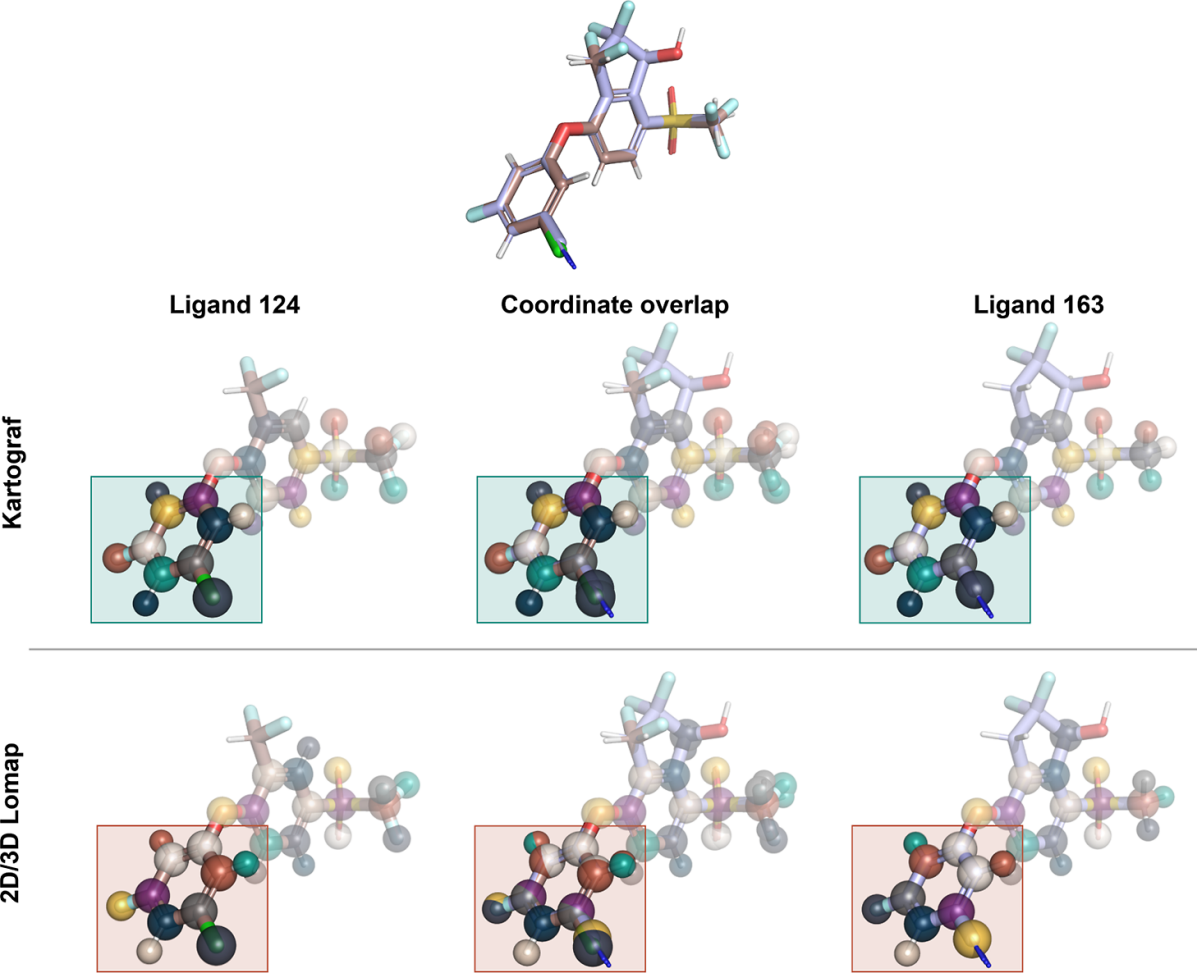
Mapping of the HIF2A ligands 124 and 163 demonstrates the differences in outputs from each mapper. Given the input coordinate set (top), the Kartograf mapper (middle) maps atoms such that the spatial distance is minimal (colored spheres). However, in this case, the Lomap mapping approaches (bottom) “realigned” the mappings, such that in the final hybrid coordinates, the ring was inverted (colored spheres). Such a “realignment” could potentially lead to an undesired alternative binding mode simulated in the free energy calculations.

Comparing the number of mapped atoms for the RHFE, TYK2, and HIF2A data sets, we found that in most cases, the atom mappers generated mappings of equal amounts of atoms being mapped (see Figures S3–S5). In a limited number of cases, Kartograf generated mappings with fewer matched atoms for mappings: RHFE data set 3% (5 mappings), TYK2 data set 2% (5 mappings), and HIF2A 1% (12 mappings). A visual inspection of the exceptional cases showed a few reasons for the outliers. In a substantial number of cases, the SIS-based approaches “realigned” the ligands to maximize the number of mapped atoms (see Figures 7 and S6). This was quantified by counting the number of mappings that displaced more than 3 atoms with a distance larger than 1.2 Å. Of the total 1755 mappings, the 2D Lomap approach “realigned” 891 mappings and the 3D Lomap approach “realigned” 310 mappings, but no mapping was observed to be “realigned” in the Kartograf’s atom mapping approach. In one case of the RHFE data set, it was found that the ligand alignment was nonoptimal, leading to a decreased number of atoms mapped by Kartograf (see Figure S7). This finding emphasizes the importance of the molecule input coordinates for Kartograf’s atom mapper.

**Figure 8:**
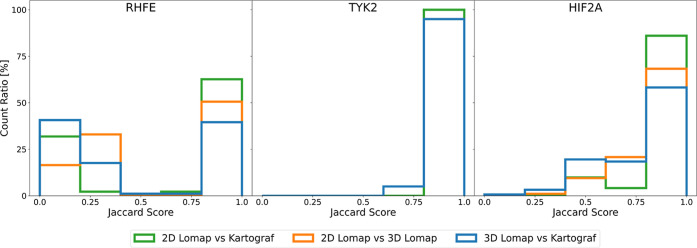
Jaccard score histograms illustrate the diversity between two mapping approaches. The Jaccard score compares two mappings by evaluating their selection of atom pairs. A score of 1 indicates identical mappings, while a score close to 0 suggests that the mappings consist of entirely different atom pairs. The comparisons are presented here for each data set and every combination of atom mapper used.

In the next step, the diversity of mappings between the three different mappers was investigated by using the Jaccard score (see Figure 8). If the Jaccard score is 1 between two mappings, then both mappings are equal. If the score is not close to 1, then mappings do not share the same mapped atom pairs. The RHFE data set was the most diverse set of all mappings. 50% of mappings were shared between 3D Lomap and Kartograf, 60% were identical between 2D Lomap and 3D Lomap, and 70% were identical between 2D Lomap and Kartograf. We note that the molecules in the RHFE data set have a very small number of atoms. As a consequence, small changes in the mapping lead to very large differences in the Jaccard score. The three different mapping approaches generated the least diverse mappings in the TYK2 data set, where 95% of the mappings were identical across the different mapping approaches. Finally, in the HIF2A set, the mappings generated using the different approaches were again more diverse. Here, Kartograf shared 60% of identical mappings with 3D Lomap and 80% with 2D Lomap. Between 2D Lomap and 3D Lomap, 65% of mappings were shared. In very low Jaccard scores (i.e., very diverse mappings), the SIS approaches often “realigned” the molecules to map them. Such “realignments” could lead to larger changes, translating or rotating substructures and effectively modifying a given binding mode (see Figure 7).

As a summary of this first analysis, we considered most mappings from the three different mapping approaches to be similar. Still, we observed a non-negligible portion of diverse mappings, which mainly were derived from so-called “realignments” in which atom positions were shifted in the mapping structures by the SIS approaches, ignoring the geometry.

### Investigating Mappings with Mapping Scores

**Figure 9:**
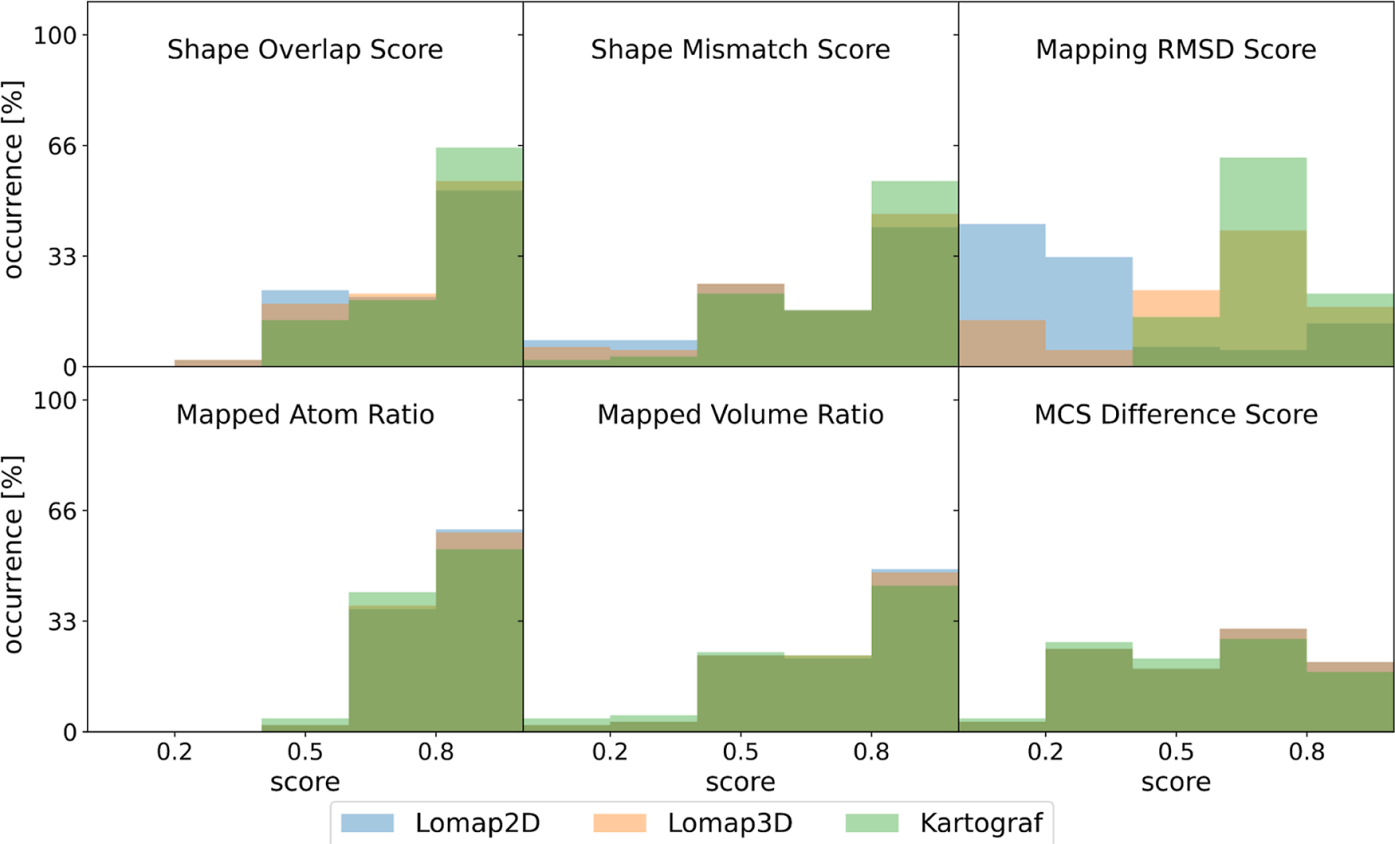
Application of mapping scores to all generated mappings is summarized in histograms (2D Lomap in blue, 3D Lomap in orange, and Kartograf in green). The score ranges from 1, a “good” score, to 0, a “bad” score. There is generally a high overall overlap in the scores, with the exception of the mapping RMSD score. For the mapping RMSD scores, the two Lomap approaches generated mappings with lower scores.

We next analyzed each mapping using six distinct scores: SOS, SMS, the mapping RMSD score, MAR, MVR, and the MCS difference score. Generally, for all mapping scores, large histogram overlaps were observed using the different mapping methods, hinting at very similar score performance, except for the mapping RMSD score. The mapping RMSD score showed a lower performance with the Lomap approaches compared to that of the Kartograf’s atom mapper (see Figure 9). This performance difference finding might not come as a large surprise as Kartograf focuses on finding an optimal solution to RMSD differences.

In the next step, it was aimed to determine if the very diverse cases found with the Jaccard scores could be explained using a single mapping score for one of the two mappings. Upon calculating the correlation between all Jaccard scores and the different mapping scores applied to all mappings of one method, we observed poor correlation across all mapping metric scores toward the Jaccard score (with *r*_Pears_ < 0.35 and τ_Kendall_ < 0.25), except for the mapping RMSD score. In the case of the mapping RMSD score vs the Jaccard score, an overall *r*_Pears_ correlation of 0.95 and 0.82 was found for 2D Lomap and 3D Lomap, respectively (τ_Kendall_ of 0.84 and 0.69). For Kartograf mappings, the mapping RMSD score vs the Jaccard score correlation was relatively poor, with an *r*_Pears_ of 0.47 and a τ_Kendall_ of 0.34. This outcome was anticipated as the core of the Kartograf atom mapper algorithm is based on an algorithm that finds the optimal atom mapping concerning geometric distances, while the Lomap approaches do not prioritize this aspect.

In terms of correlations between different mapping scores, we identified strong correlations between the two shape-based scores ( and ) and a moderate correlation with the RMSD score (SMS  and , SOS:  and ). A strong correlation was found between MVR and MAR ( and ), with a weak correlation to the RMSD mapping score ( and ). Another strong correlation of  and  was observed between MVR and the MCS difference score. Interestingly, an anticorrelation was detected between SMS and SOS versus MVR, MAR, and the MCS difference score (*r*_Pears_ < −0.5 and τ_Kendall_ < −0.5), indicating a partially mutually exclusive relationship.

In conclusion, the analysis of the mapping scores identified three correlating groups: the first group comprises SOS and SMS, the second group includes MVR, MAR, and the MCS difference score, and the final group consists solely of the mapping RMSD score. Concerning the mapping diversity found in the Jaccard scores, a high correlation of the mapping RMSD score with mappings of the Lomap approaches was observed. Further visual examination of the RMSD outliers revealed the previously mentioned “realignments” as the primary cause of low scores. The interpretation of the score depends on the expectations for the coordinates as it evaluates how close the final mapping coordinates are in comparison with the input. If the given coordinates originate from, for example, a ligand-based approach without any ligand–protein complex information, then the RMSD deviations detected could be considered an improvement to the coordinates. However, in a case where the molecules were modeled in an exact desired binding mode, the score indicates a deviation from that binding mode, which should be avoided. The MVR differences were mainly triggered by Kartograf for the RHFE data set due to the fewer mapped atoms observed in the comparative analysis of the number of mapped atoms. A visual inspection of the results shows that shape-based mapping scores picked up large shape changes in the mappings and input alignment deviations (which might lead to a shape overlap decrease in the hybrid-topology approach). These mapping scores could be an attractive addition to scoring the complexity of a transformation with given binding modes. As a final remark, future work may select one mapping score of the highly correlating score groups to represent different aspects in a mapping evaluation.

### Time Consumption Comparison of Mapping Approaches

As anticipated in the Introduction, it was found that the time required to generate the mappings is directly proportional to the combination of the number of states and the number of atoms involved in a mapping. This means that as the complexity of the system (in terms of both states and atoms) increases, the time required to create the mappings also increases.

The three atom mapping methods required the same amount of time of 3 s for the all-to-all mappings of the RHFE set, which translates to 91 mappings with an average of 38.6 atoms per mapping. However, for TYK2 with 120 all-to-all mappings and an average atom count in the mappings of 74, the time difference reaches 18 s between the Lomap approaches taking 26 s and the Kartograf approach taking 8 s.

For the HIF2A data set, with 666 mappings with an average of 73 atoms per mapping, the difference between the methods exceeded 10 min. The 3D Lomap approaches required more than 11 min, while the Kartograf approach resolved the all-to-all mappings in approximately 1 min.

As an extreme example, we also attempt to generate a mapping from a full protein mutation transformation of ASP to TYR in TYK2 with 9349 atoms. Here, Kartograf solved a mapping within 2 s, while the SIS-based algorithms were unable to converge to a solution within a 4 h limit (see Table 1). It is noted that the Lomap approaches with the isomorphic graph search were designed with small molecules in mind.

**Table 1:** Mapping Time Consumption Comparison among 3D Lomap, 2D Lomap, and Kartograf across Diverse Input System Complexities, Varying the Number of States and the Number of Atoms^a^

system	*n*_states_	avg 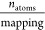	mapper	gen. mappings	duration
RHFE	14	38.6	Kartograf	182	3 s
			3D Lomap		3 s
			2D Lomap		3 s
TYK2	16	74.0	Kartograf	240	8 s
			3D Lomap		26 s
			2D Lomap		26 s
HIF2A	35	73.0	Kartograf	1225	58 s
			3D Lomap		11 min 33 s
			2D Lomap		11 min 16 s
protein	2	9349.0	Kartograf	1	2 s
			3D Lomap		>4 h
			2D Lomap		>4 h

a It should be noted that Lomap was not designed to tackle protein mutation challenges, yielding an unfair comparison.

### Calculation of Relative Free Energies

To evaluate the atom mapping algorithm of Kartograf, we applied it along with two variants of the Lomap atom mappers to multiple relative free energy calculation approaches.

### Calculation of RHFEs

In this study, we used a toy system to calculate the RHFEs. However, we had to exclude ring-size changes from the analysis as the hybrid-topology approach did not support bond-breaking during the transformations. Initially, the same radial layout from Ries et al. was used to assess the performance of the mapping algorithms; later, an MST approach was studied.^28^

For the radial network, all of the mapping approaches successfully lead to the calculation of the necessary 13 edges to obtain the complete ranking of compounds. Comparing the generated free energies from the different starting points of Kartograf, 2D Lomap, and 3D Lomap with the experimental data, we observed no significant differences in terms of RMSE, MUE, ρ_Pears_, and τ_Kendall_ between the different approaches (see Table 2). However, two clear outliers were observed in the ΔΔ*G* correlation plots (see Table S1 and Figure S8). These outliers correspond to the ligand transformations 12–8 and 12–5, which consistently showed high deviations from the experimental values in all approaches. These deviations could potentially be attributed to sampling issues in the hybrid-topology approach, particularly for the complex transformations involving ligand 5, which were already identified as difficult in the previous work.^28^ Similarly, for transformations 12–8, the presence of a flexible and long aliphatic chain in ligand 8 may be causing sampling difficulties. An additional analysis of ring hybridization changes and the resulting torsion distributions of ligand transformations 12–2 are provided in the Supporting Information (see Section S2.1.1 and Figure S9).

**Table 2:** ΔΔ*G* RHFE-Statistics Summary of Estimate Errors and Correlation Metrics to Experiment^103^ for Each Mapping Method and Network Type^a^

	radial			MST		
network layout approach	Kartograf	2D Lomap	3D Lomap	Kartograf	2D Lomap	3D Lomap
RMSE (kcal/mol)	1.21 ± 0.21	1.22 ± 0.22	1.20 ± 0.21	1.12 ± 0.14	1.06 ± 0.17	1.28 ± 0.18
MUE (kcal/mol)	1.00 ± 0.19	1.00 ± 0.20	1.00 ± 0.19	0.97 ± 0.16	0.92 ± 0.15	1.13 ± 0.17
ρ_Pears_	0.91 ± 0.05	0.91 ± 0.05	0.92 ± 0.05	0.95 ± 0.03	0.95 ± 0.03	0.92 ± 0.05
τ_Kendall_	0.81 ± 0.10	0.76 ± 0.12	0.77 ± 0.12	0.80 ± 0.13	0.82 ± 0.13	0.76 ± 0.13

a Errors in statistical estimates are calculated via bootstrap resampling.

**Figure 10:**
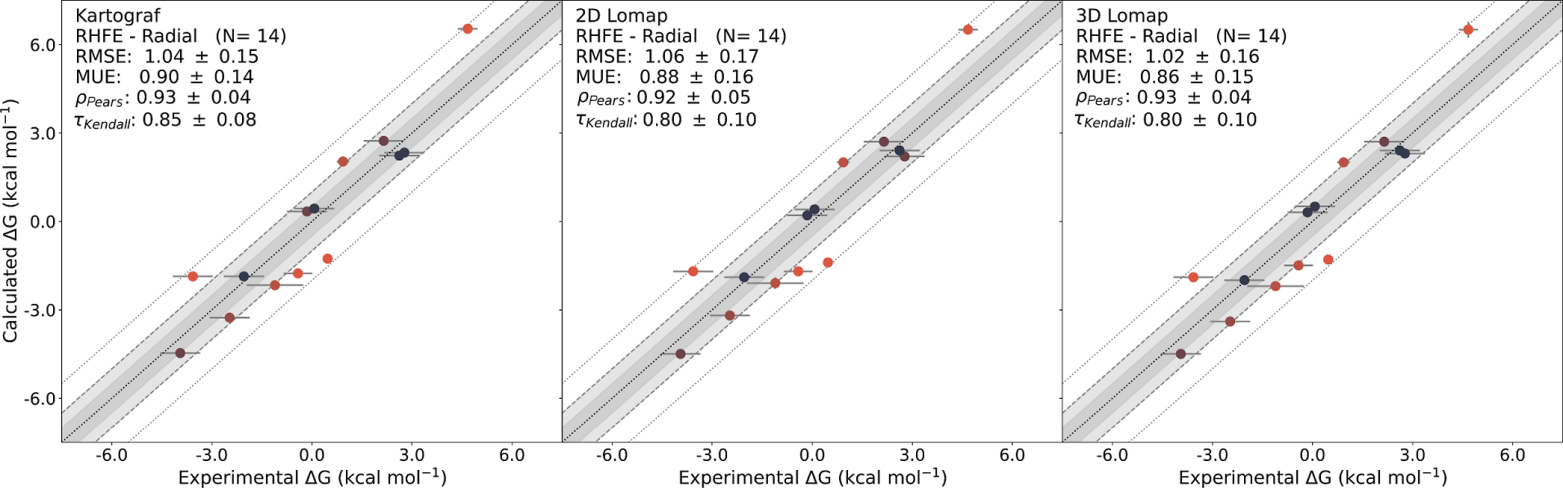
MLE-derived ABFEs for the radial RHFE data set plotted against the experimental results. A strong correlation was observed for all approaches with the experiment. The color gradient of the data points (blue: up to ≤0.5 kcal/mol; red: max. ≥2 kcal/mol) indicates the distance of the predictions from the experimental reference.

Using Cinnabar’s maximum likelihood estimation (MLE) approach, absolute Δ*G* values were predicted. The resulting statistical metrics showed trends similar to those of the ΔΔ*G* values (see Figure 10).

To explore the impact of different mappers on the network layout, we used a minimal spanning tree-ligand network layout instead of a radial map.

The usage of different mappers resulted in changes in the graph structure (Figure S10). Out of the 13 edges required to describe the complete ranking of all molecules, 5 edges were common across all networks. Two additional edges were shared between 3D Lomap and Kartograf, suggesting a closer relationship between the mappings in the data set for these two approaches.

With the MST networks, a slight but within-error improvement in observed statistics comparing the calculated values with the experimental values is seen (see Table S2, Figures S11, and S12). In all MST approaches, only one transformation edge (ligands 3–11) selected by the 3D Lomap atom mapper approach deviated more than 2 kcal/mol in all three replicates from the experimental result, leading to an average MUE of 2.55 kcal/mol.

Similar to ΔΔ*G*, the overall Δ*G* value performance of the approaches remained comparably equal, with no ligand deviating by more than 2 kcal/mol from the experimental values (see Figure S12).

### Calculation of RBFEs

Multiple RBFE campaigns were conducted to assess the performance of the three different mapping approaches in more complex scenarios.

### RBFE Calculations with the TYK2 System

We first investigated the TYK2 system, which consists of relatively small alchemical modifications (see Figure 6). The radial network layout for the 16 ligands was centered around ligand *ejm31*, resulting in the best average Lomap score across all edges. MST networks were generated for all three mappers with 14 edges. Six common edges were found across the networks, with two extra edges shared between Kartograf and 2D Lomap and six extra edges shared between Kartograf and 3D Lomap. 2D Lomap and 3D Lomap shared one exclusive edge. One unique edge was found by 3D Lomap and five by 2D Lomap for the MST network layout. As previously found, we find that the overlap in identified edges is larger between Kartograf and 3D Lomap.

Comparing the obtained ΔΔ*G* values from the different approaches revealed no significant differences between them. Interestingly, in the case of TYK2, the average statistics seemed to worsen with the MST layout compared to the radial layout, although this change was not statistically significant (see Tables 3, S3, and S4). None of the approaches yielded any outliers exceeding 2 kcal/mol deviation from the experimental values (see Figures S14 and S15).

**Table 3:** ΔΔ*G* TYK2 RBFE-Statistics Summary of Estimate Errors and Correlation Metrics to Experiment^103^ for Each Mapping Method and Network Type of the TYK2 System^a^

	radial			MST		
network layout approach	Kartograf	2D Lomap	3D Lomap	Kartograf	2D Lomap	3D Lomap
RMSE (kcal/mol)	0.65 ± 0.13	0.69 ± 0.15	0.65 ± 0.14	0.90 ± 0.17	0.79 ± 0.14	0.92 ± 0.15
MUE (kcal/mol)	0.53 ± 0.11	0.54 ± 0.12	0.48 ± 0.12	0.72 ± 0.16	0.64 ± 0.13	0.76 ± 0.16
ρ_Pears_	0.72 ± 0.13	0.74 ± 0.11	0.78 ± 0.11	0.41 ± 0.29	0.65 ± 0.18	0.40 ± 0.29
τ_Kendall_	0.46 ± 0.18	0.57 ± 0.16	0.50 ± 0.17	0.14 ± 0.18	0.41 ± 0.20	0.21 ± 0.20

a Errors in statistical estimates are calculated via bootstrap resampling.

**Table 4:** ΔΔ*G* HIF2A RBFE-Statistics Summary of Estimate Errors and Correlation Metrics to Experiment^109^ for Each Mapping Method and Network Type of the HIF2A System^a^

	radial			MST		
network layout approach	Kartograf	2D Lomap	3D Lomap	Kartograf	2D Lomap	3D Lomap
RMSE (kcal/mol)	1.96 ± 0.18	2.16 ± 0.22	2.05 ± 0.22	1.58 ± 0.30	1.97 ± 0.29	1.59 ± 0.36
MUE (kcal/mol)	1.67 ± 0.18	1.81 ± 0.20	1.73 ± 0.19	1.20 ± 0.19	1.54 ± 0.21	1.17 ± 0.20
ρ_Pears_	0.33 ± 0.12	0.26 ± 0.13	0.25 ± 0.15	0.29 ± 0.22	0.15 ± 0.21	0.21 ± 0.31
τ_Kendall_	0.26 ± 0.11	0.22 ± 0.12	0.18 ± 0.12	0.29 ± 0.12	0.18 ± 0.14	0.22 ± 0.14

a Errors in statistical estimates are calculated via bootstrap resampling.

**Figure 11:**
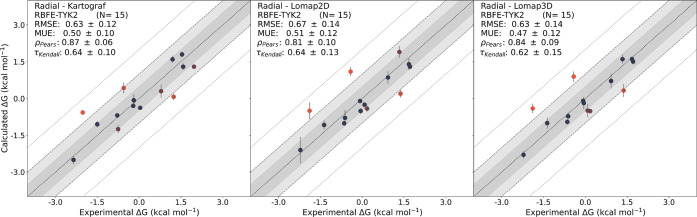
MLE-derived ABFEs for the radial TYK2 data set plotted against the experimental results. The color gradient of the data points (blue: up to ≤0.5 kcal/mol; red: max. ≥2 kcal/mol) indicates the distance of the predictions from the experimental results.

To evaluate the overall performance of the approaches, we compared the Δ*G* values that did not exhibit any significant changes between the three approaches (see Figure 11 and Table S4).

### RBFE Calculations with the HIF2A System

Lastly, the HIF2A system with 35 ligands was used as a test system with changes in multiple regions of the ligands (see Figure S1).

The radial network layout was formed around ligand 163, which yielded a radial network with the best average Lomap score across all edges.

MST networks containing 34 transformations were generated for all three mapping methods; of these, 18 edges were shared across all three networks. Kartograf and 2D Lomap shared two additional edges; Kartograf and 3D Lomap shared 11 exclusive edges; and 2D Lomap and 3D Lomap shared five additional edges. The network constructed with Kartograf identified two unique edges, while 2D Lomap identified nine unique edges.

**Figure 12:**
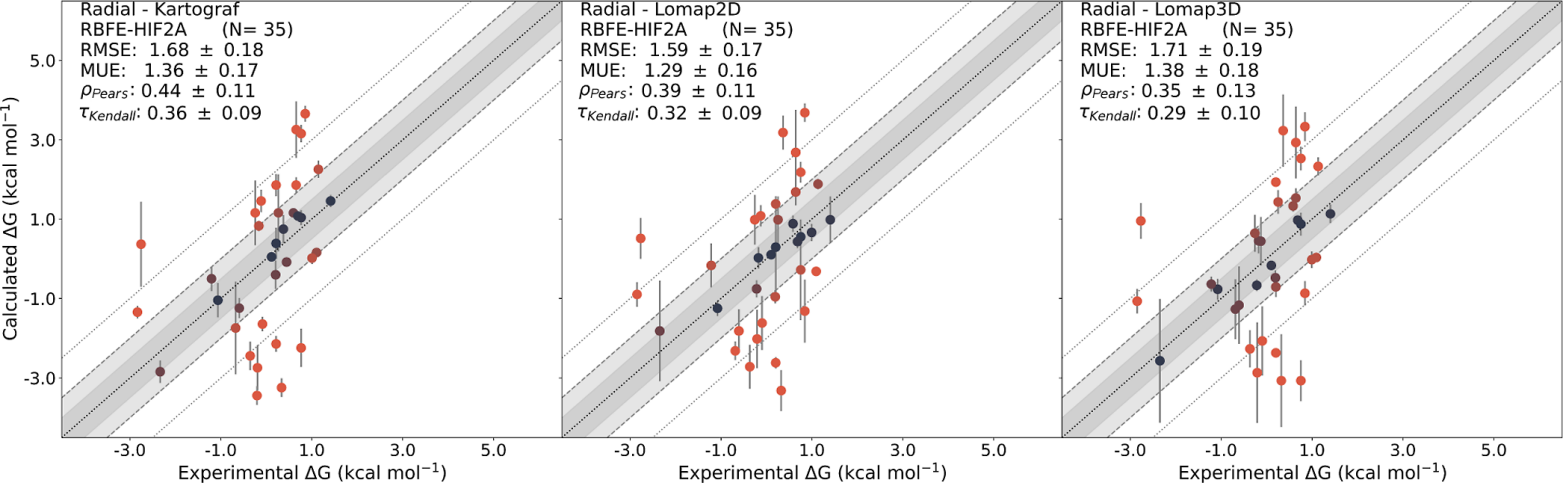
MLE-derived ABFEs for the radial HIF2A data set plotted against the experimental results. The color gradient of the data points (blue: up to ≤0.5 kcal/mol; red: >2 kcal/mol) indicates the distance of the free energy calculation results from the experimental results.

Once again, all approaches yielded comparable results without any significant differences being detected. However, in the HIF2A data set, the average statistics of the MST approach showed slight improvements in RMSE and MUE compared to the radial network layout. Nevertheless, these observations were not significant. Based on the analysis of the HIF2A data set, we can conclude that all three approaches (Kartograf, 2D Lomap, and 3D Lomap) demonstrate comparable performance. Despite the complexity of the data set, none of the approaches showed a significant advantage over the others in terms of ΔΔ*G* performance (see Figure 12, Table 4, Tables S5 and S6, and Figures S16–18).

### Torsion Sampling Analysis of Ligand 163 in the Context of “Realignments” in Mappings

To investigate the impact of “realignments” by the Lomap mapping approaches that inverted the coordinates of a benzyl substituent, potentially impacting the binding mode during simulations, a torsion analysis was conducted for molecule ligand 163. The analysis focused on the torsional distributions of all heavy-atom-related dihedrals in both water and complex simulations.

**Figure 13:**
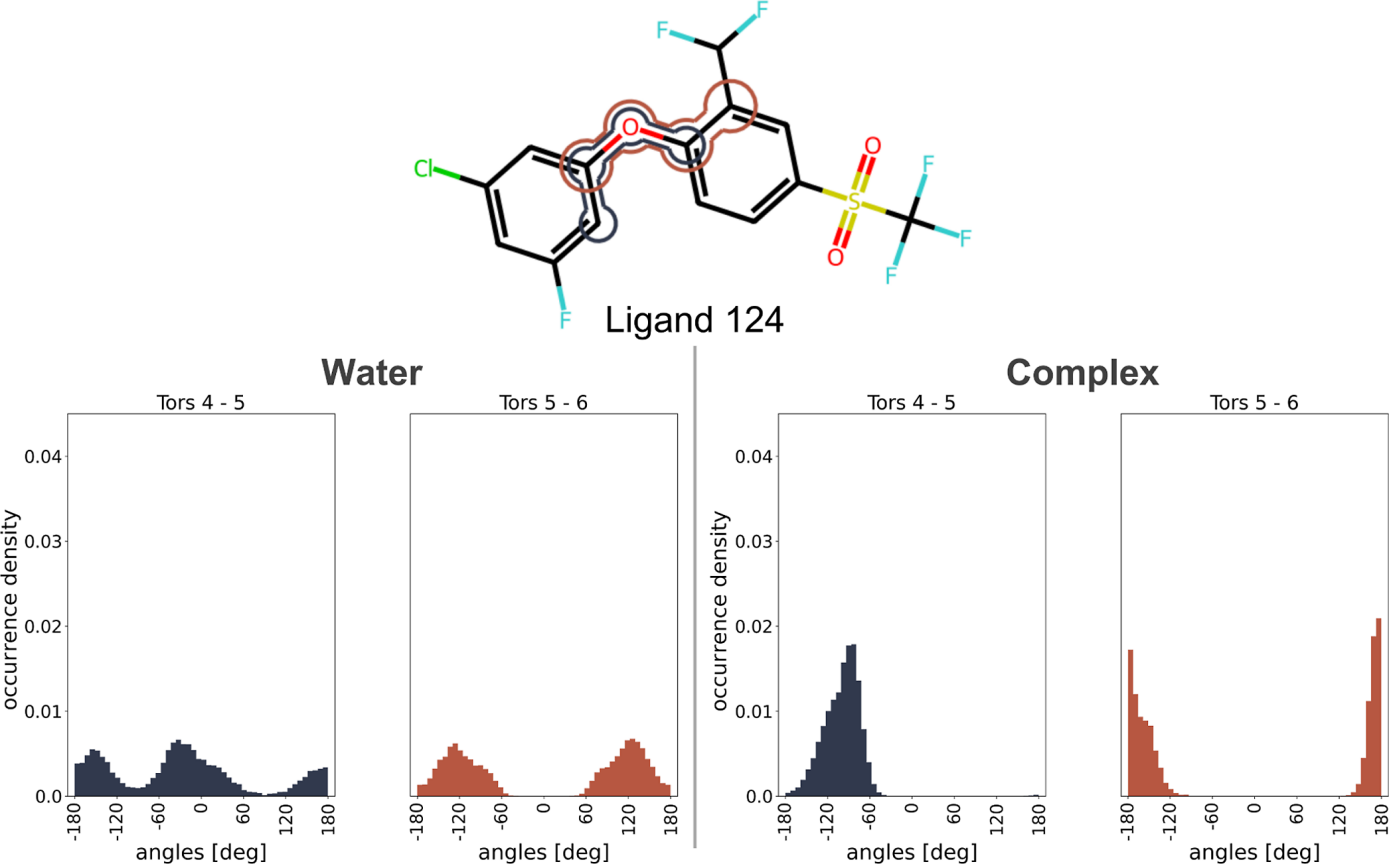
Torsion angle (Tors) distributions of ligand 124 sampled during the simulation. These results indicate that the rotation around Tors 4–5 and Tors 5–6 can occur in the water environment but not in the complex. Combined with our findings in 7, this indicates the possibility for these realignments to lead to sampling issues should they not be resolved during system equilibration.

In the water case, it was observed that the bonds between atoms 4–5 and 5–6 were able to rotate fully during the simulations. However, in the complex environment with HIF2A, the behavior changed due to the steric hindrance of the protein. No rotation of the ring was observed in any of the three replicates (Figure 13). This suggests that for the atom mapping generated by 2D Lomap and 3D Lomap for this ligand pair, the binding mode might be altered (see Figure 7). In the simulated case, we found that the correct orientation was obtained on energy-minimizing the system, and therefore no change of the binding mode occurred, fortunately.

This finding emphasizes the importance of carefully considering atom mappings, especially for ligand pairs in complex environments. Ensuring that the input coordinates accurately represent the desired binding mode in a 3D coordinate system is crucial to avoid potential issues during simulations.

## Conclusions

We have introduced Kartograf, a tool that offers an efficient algorithm for atom mappings based on 3D coordinates. This algorithm is suitable for both small molecules and potentially large biomolecules. Kartograf also provides useful features such as 3D-based mapping scores that could be further used in novel atom mapping scoring functions.

The evaluation of the atom mapper showed that Kartograf contains a fast mapping approach for a large number of molecules and a large number of atoms contained in their mappings, often yielding similar mapping results compared to those of Lomap. If the coordinates should be preserved as much as possible, we want to suggest Kartograf’s atom mapper as a tool of choice. However, for Kartograf’s mapping algorithm, the alignment of the ligands is crucial, and a possible source of error was found. To potentially overcome alignment problems, Kartograf offers shape and SIS alignment wrapping functions. Additionally, we want to mention that there are a plethora of alternative MCS finding algorithms, as described, for example, by Raymond and Willett, which could be further compared to the here presented atom mapper in order to get a better understanding of the advantages and disadvantages of our approach.^54^

To evaluate the performance of Kartograf’s atom mapper, multiple atom mappers and various network layouts were compared using the RFE protocol approach of OpenFE. Our results showed that the Kartograf algorithm produced comparable results to the Lomap atom mappers for the given test sets. In the future, we would like to continue to monitor the performance of Kartograf in the context of more diverse and larger benchmark sets, with the expectation of a much faster mapping phase and smaller geometric perturbations derived from the atom mappings for the calculations.

Kartograf’s mapping algorithm may also be useful for other applications that require mappings, such as product/educt mapping in reaction predictions with QM software or the protein mutation use case.

The presented geometry-based mapping scores, especially the mapping RMSD score, could show potential in the development of future mapping scorers.

The code for Kartograf is available on GitHub (https://github.com/OpenFreeEnergy/kartograf) and can be easily used within the OpenFE environment or as a standalone package, allowing for good interoperability between different packages. Installation as a standalone process can be performed using pip and conda.

## Data Availability

The code of Kartograf is available at https://github.com/OpenFreeEnergy/kartograf. For the relative free energy simulations, we used OpenFE, which is available at https://github.com/OpenFreeEnergy/openfe. The RHFE, TYK2, and HIF2A System start coordinates can be accessed at https://github.com/OpenFreeEnergy/openfe-benchmarks.

## Supporting Information Available

Additional descriptions of the HIF2A and protein mutation systems, correlation plots of the number of mapped atoms in test networks, visualization of edge case mappings, analysis of the sampled torsion distributions of ligand 2 from the RHFE data set, and individual free energy calculation results and additional correlation plots for the radial and MST networks (PDF)
