# Supplementary material for: Kartograf: A Geometrically Accurate Atom Mapper for Hybrid-Topology Relative Free Energy Calculations
Source: J Chem Theory Comput. 2024 Feb 8;20(5):1862–77. doi: 10.1021/acs.jctc.3c01206 (PMC10941767; doi:10.1021/acs.jctc.3c01206)
Supplement: Supplementary file 1 — ct3c01206_si_001.pdf [file ct3c01206_si_001.pdf]

SUPPORTING INFORMATION
Kartograf: A Geometrically-Accurate Atom Mapper for Hybrid Topology Relative Free Energy Calculations

Benjamin Ries,[ab], Irfan Alibay,[b], David WH Swenson,[b] Hannah M Baumann[b] Mike M Henry,[bc] James RB Eastwood[b], and Richard J Gowers,[b]

[a] *Boehringer Ingelheim Pharma GmbH & Co KG, Medicinal Chemistry, Birkendorfer Str 65, 88397 Biberach an der Riss, Germany*
[b] *Open Free Energy, Open Molecular Software Foundation, Davis, CA, 95616, United States.*
[c] Computational and Systems Biology Program, Sloan Kettering Institute, Memorial Sloan Kettering Cancer Center, New York, NY, 1275, USA
E-mail: benjamin.ries@boehringer-ingelheim.com

Contents

1 Additions to Theoretical Analysis **2**
1.1 Systems . . . 2
1.1.1 HIF2A . . . 2
1.1.2 Protein Mapping . . . 3
1.2 Mapping Comparisons . . . 4
1.3 Mapping Examples . . . 7

2 Additions to RHFE Simulations **8**
2.1 Radial Networks . . . 8
2.1.1 Aliphatic/Aromatic Rings . . . 9
2.2 MST Networks . . . 11

3 Additions to RBFE Simulations **13**
3.1 TYK2 . . . 13
3.1.1 Radial Networks . . . 13
3.1.2 MST Networks . . . 14
3.2 HIF2A . . . 16
3.2.1 Radial Networks . . . 16
3.2.2 MST Networks . . . 18

1 Additions to Theoretical Analysis

1.1 Systems

1.1.1 HIF2A

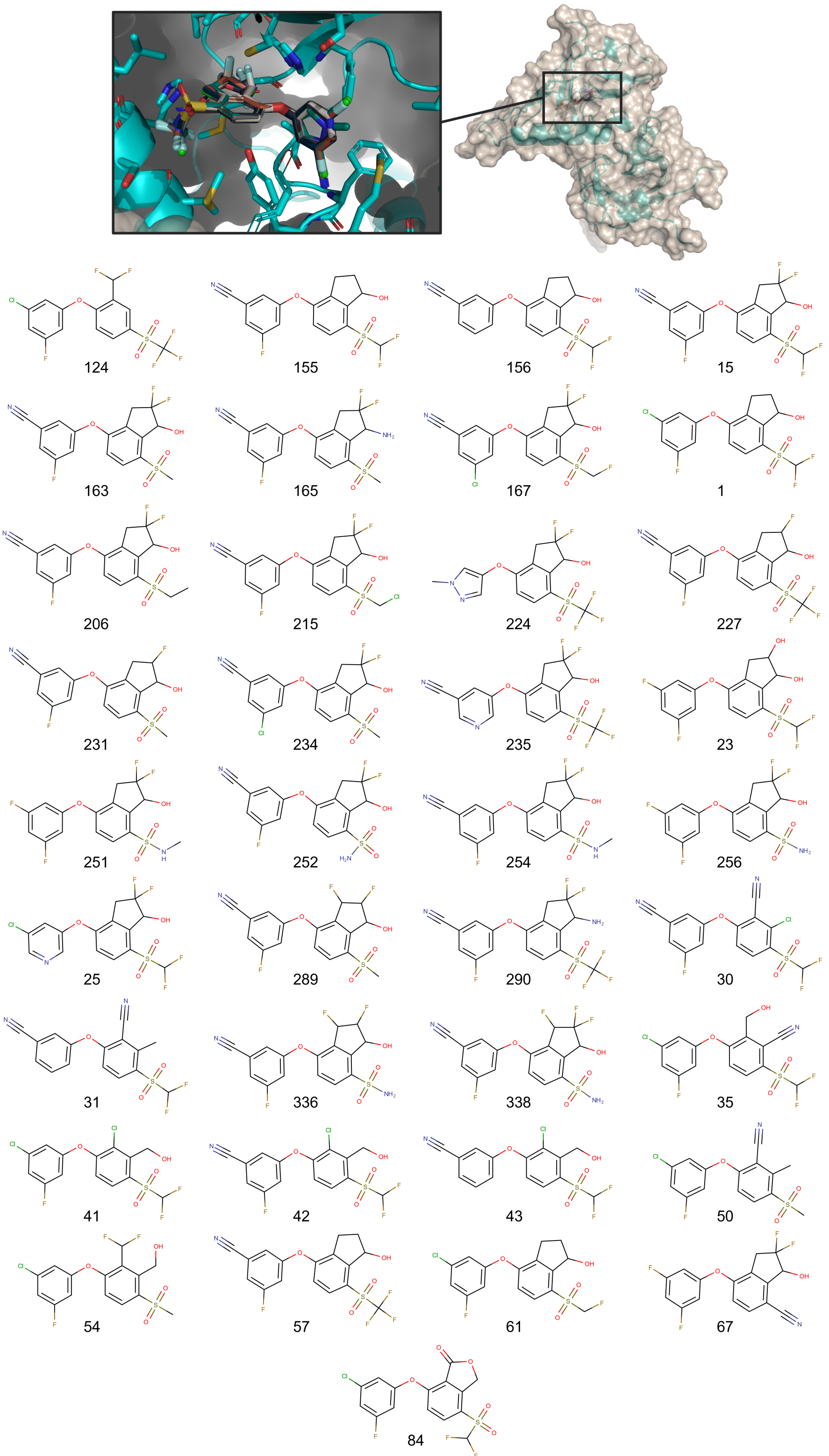

Figure S1: HIF2A System with 35 Ligands[106]

1.1.2 Protein Mapping

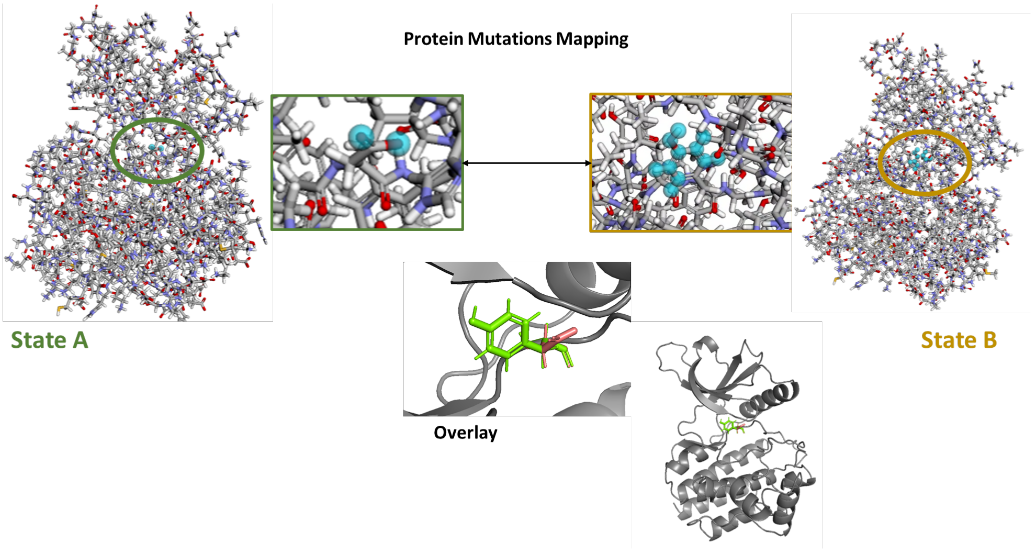

Figure S2: Protein amino acid mutation studies using free energy calculations, could be a potential application for Kartograf. As a test system TYK2 was used and Asparagine 153 was mutated to Tyrosin. The resulting mapping and the provided input structures are illustrated in the figure.

1.2 Mapping Comparisons

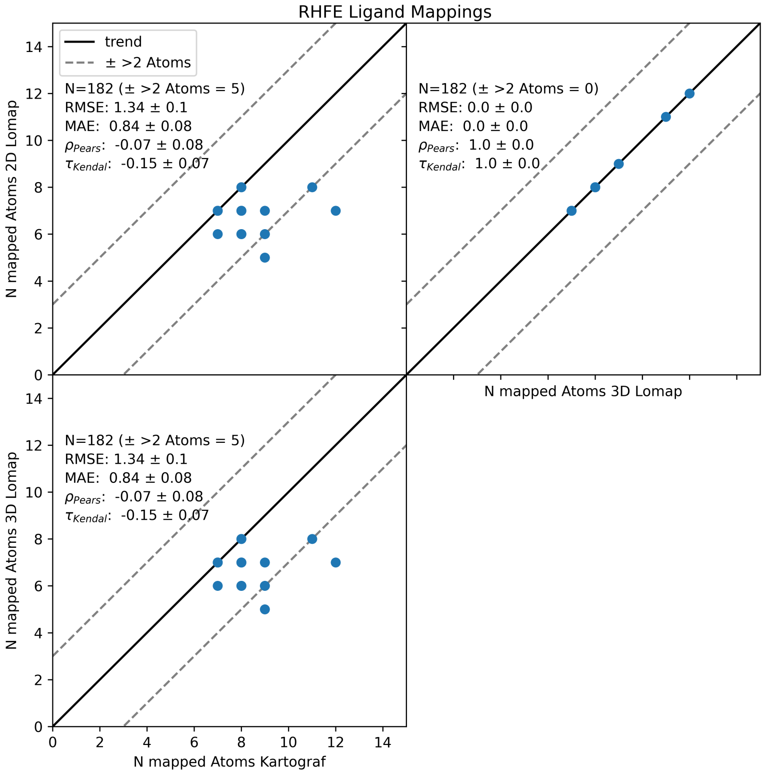

Figure S3: Comparison of the number of mapped atoms between the different mapping approaches for the RHFE system.

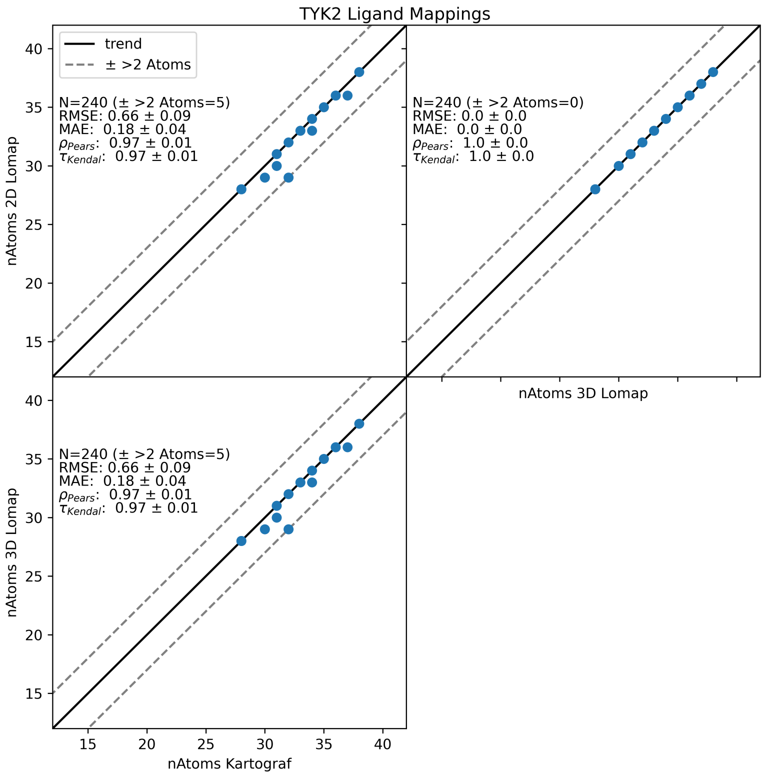

Figure S4: Comparison of the number of mapped atoms between the different mapping approaches for the TYK2 system

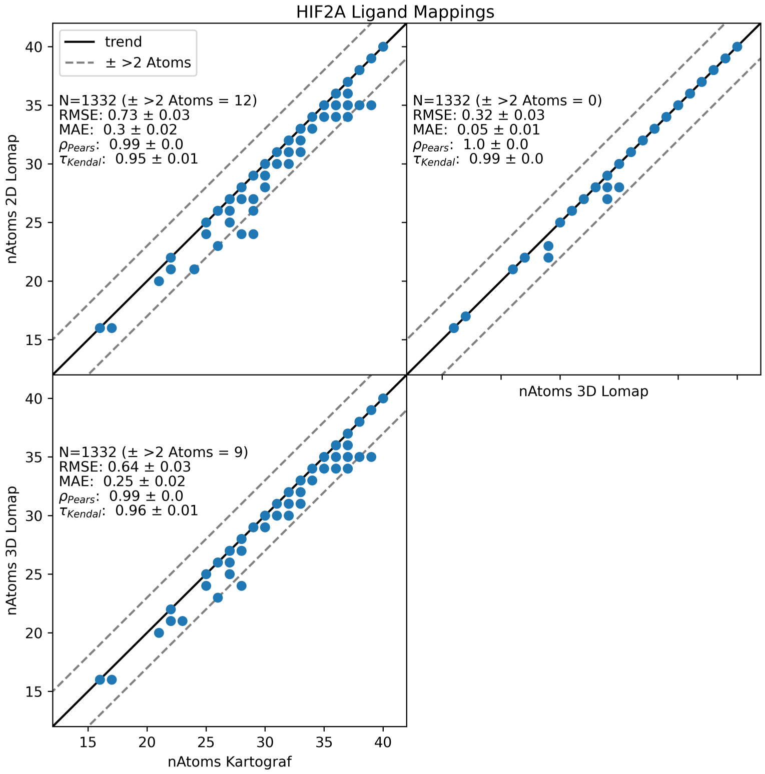

Figure S5: Comparison of the number of mapped atoms between the different mapping approaches for the HIF2A system

1.3 Mapping Examples

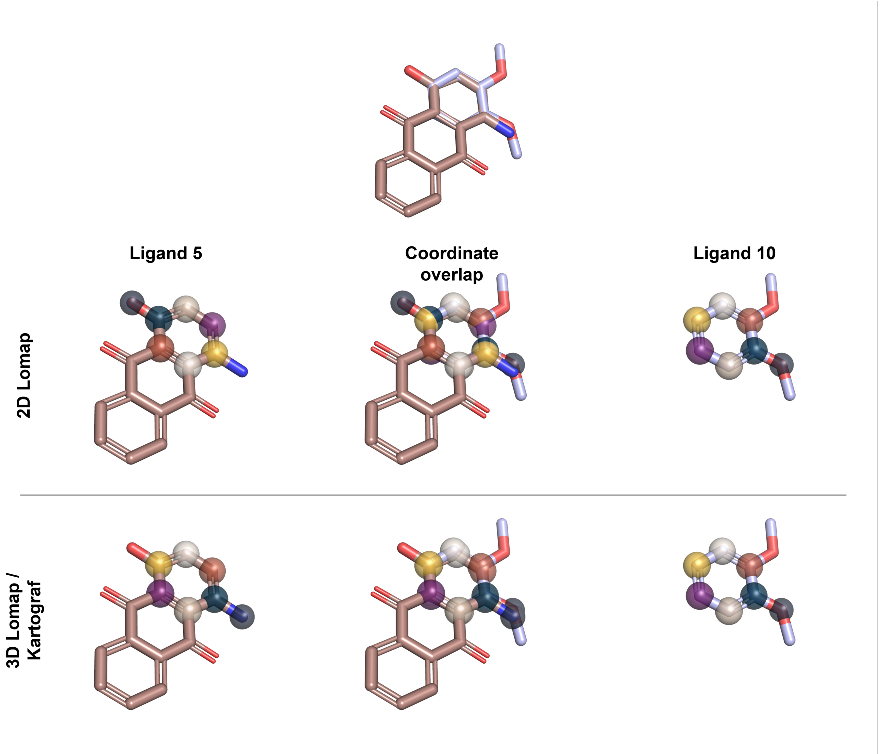

Figure S6: The presented mapping of molecule 5 (brown) and molecule 1 (light blue) contains 're-alignments' in the 2D Lomap mapping, as indicated by the colored spheres. These 're-alignments'; can not be found in the 3D Lomap and Kartograf mapping approaches.

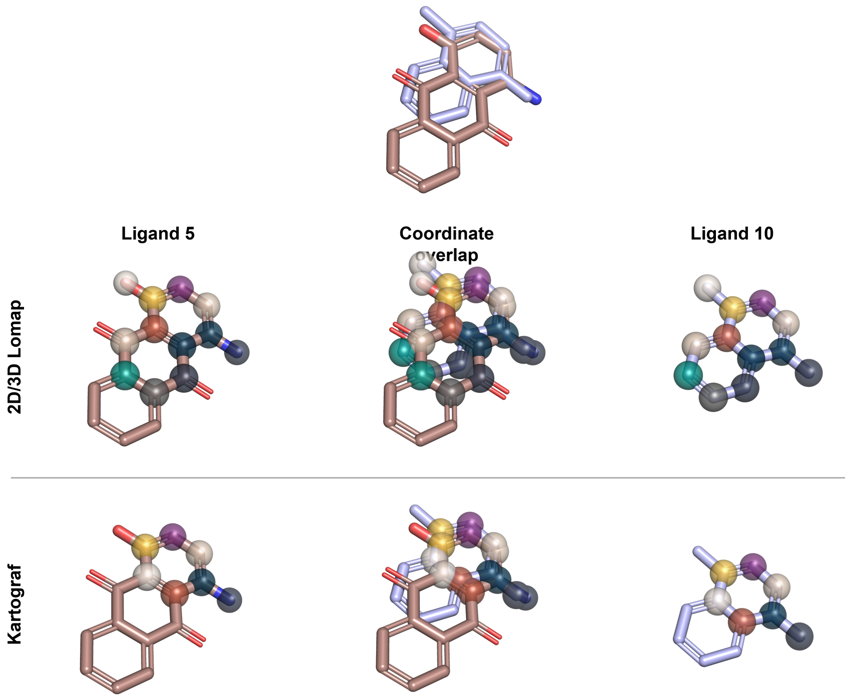

Figure S7: Molecule 5 (brown) and molecule 10 (light blue) of the RHFE ligand set, were found to be not perfectly aligned leading to a reduced number of mapped atoms in the Kartograf approach (see spheres), compared to the Lomap approaches.

2 Additions to RHFE Simulations

2.1 Radial Networks

ligA	ligB	experiment $[kcal/mol]$	$\Delta\Delta G$ Kartograf $[kcal/mol]$	$\Delta\Delta G$ Lomap2D $[kcal/mol]$	$\Delta\Delta G$ Lomap3D $[kcal/mol]$
12	6	6.72 ± 0.85	6.8 ± 0.05	6.7 ± 0.07	6.8 ± 0.08
12	16	6.1 ± 0.85	7.2 ± 0.05	7.2 ± 0.04	7.2 ± 0.08
12	8	0.38 ± 0.85	2.6 ± 0.03	2.8 ± 0.16	2.6 ± 0.08
12	10	1.91 ± 0.85	2.6 ± 0.06	2.6 ± 0.03	2.5 ± 0.02
12	9	6.57 ± 0.85	6.7 ± 0.06	6.9 ± 0.11	6.9 ± 0.05
12	15	1.48 ± 0.85	1.2 ± 0.04	1.3 ± 0.02	1.1 ± 0.06
12	5	8.62 ± 0.67	11.0 ± 0.18	11.0 ± 0.19	11.0 ± 0.29
12	14	3.8 ± 0.85	4.8 ± 0.08	4.7 ± 0.04	4.8 ± 0.03
12	3	3.53 ± 0.74	2.7 ± 0.09	2.8 ± 0.06	3.0 ± 0.08
12	2	2.84 ± 1.04	2.3 ± 0.06	2.4 ± 0.21	2.3 ± 0.07
12	11	4.88 ± 0.63	6.5 ± 0.03	6.5 ± 0.02	6.5 ± 0.13
12	1	4.42 ± 0.61	3.2 ± 0.08	3.1 ± 0.09	3.2 ± 0.12
12	13	4.02 ± 0.85	4.9 ± 0.05	4.9 ± 0.06	5.0 ± 0.05

Table S1: RHFE $\Delta\Delta G$ - Radial Network - The table shows the results of the free energy calculations for the three different mapping approaches.

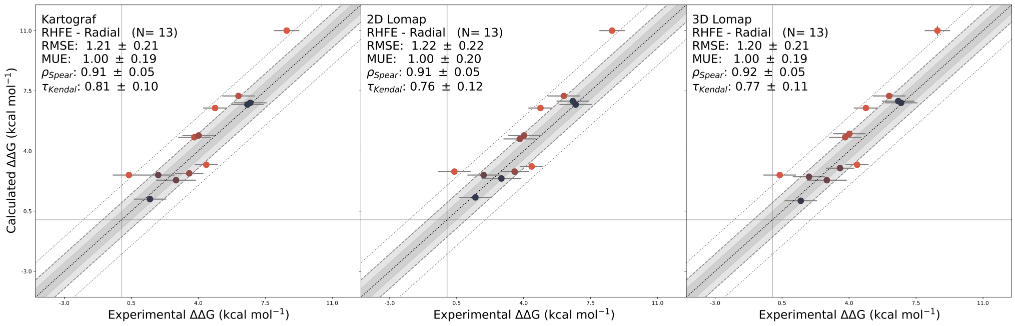

Figure S8: Relative binding free energies for the radial RHFE dataset plotted against the experimental results.

2.1.1 Aliphatic/Aromatic Rings

We also investigated the challenging transformations involving aliphatic ring to aromatic ring conversions (transformations $12-3$ and $12-2$), as previously studied by Ries et al..[28] In the case of the hybrid topology approach, we analyzed the torsional distributions in the end-state simulations of ligands 12, 2, and 3, expecting to observe a fully planar ring for the ligand 12 end-state simulation and a boat or chair configuration for the end-state simulations of ligand 2 and 3. However, upon visual inspection, we found a slightly bending aromatic ring in the ligand 12 end-state simulation and a flattened aliphatic ring in the ligand 2 and 3 end-state simulations. Three ring torsions of the ring structure were measured for the benzyl state, where 0 degree torsions are expected. In the 3D and 2D LOMAP case the average angles where $[-13.1\pm6.5, -15.0\pm5.9, 20.6\pm6.1]$, in the Kartograf approach the angles where $[-8.67\pm 12.7, -2.6\pm6.8, 1.3\pm7.2]$ (see Figure S9). These sampling issues arise from the current implementation status, which does not allow the turning off and on of torsion force field contributions in transformations. Consequently, the default for the atom mapper in Kartograf does not support such transformations.

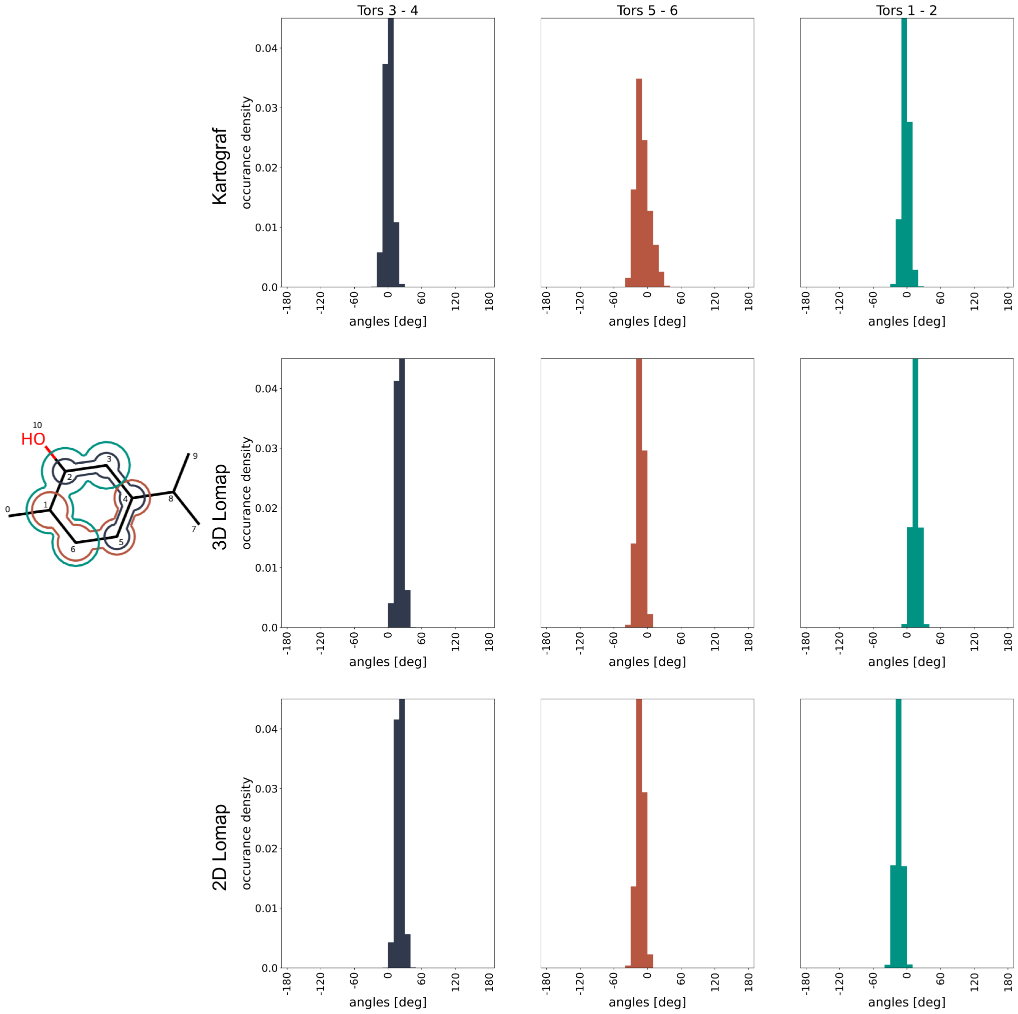

Figure S9: Torsion angle (Tors) distribution of the aliphatic molecule 2 in the sampling state of the aromatic molecule 12. The expected completely planar ring was not observed in any of the approaches.

2.2 MST Networks

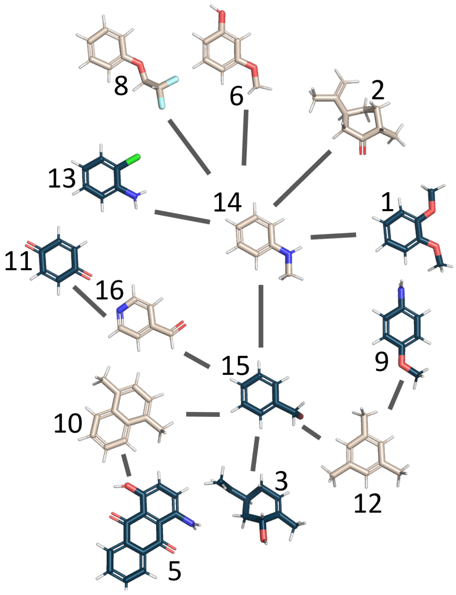

(a) Kartograf

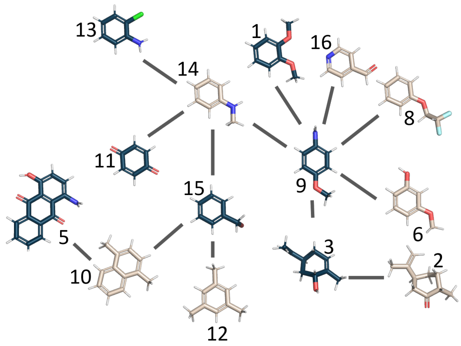

(b) 2D Lomap

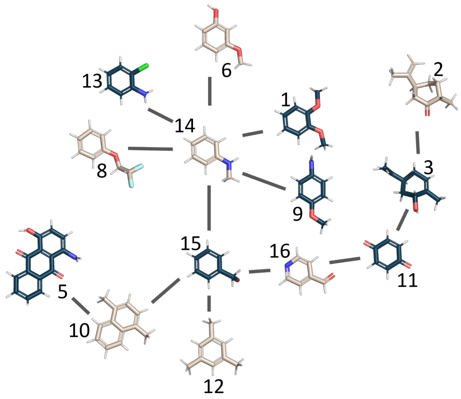

(c) 3D Lomap

Figure S10: The presented MST networks using the Lomap mapping scorer to build the network.

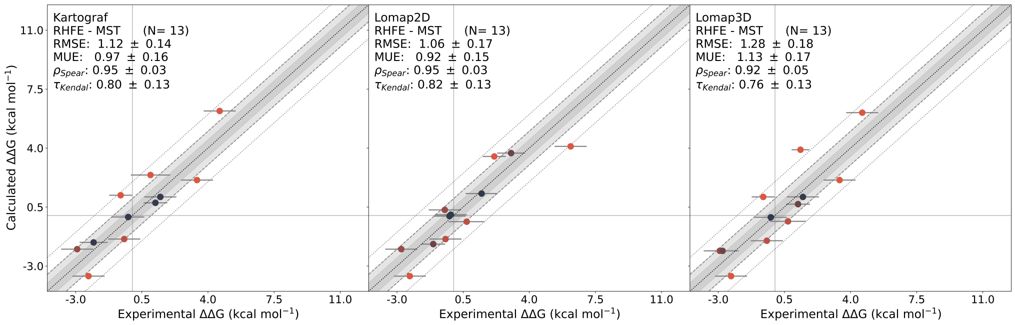

Figure S11: Relative binding free energies for the MST RHFE dataset plotted against the experimental results.

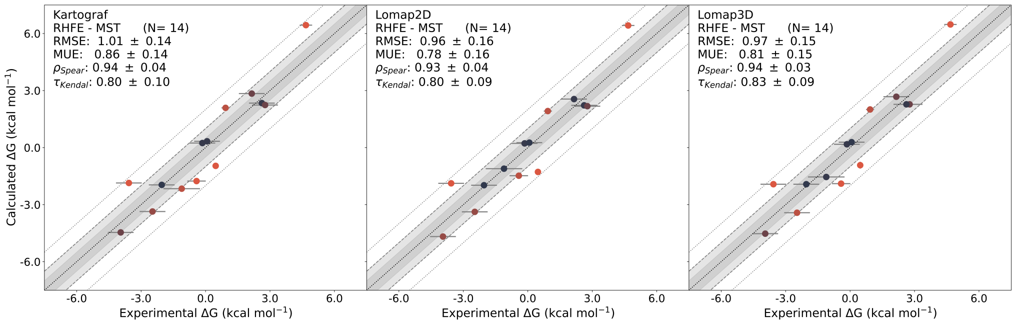

Figure S12: MLE derived absolute binding free energies for the MST RHFE dataset plotted against the experimental results.

ligA	ligB	experiment [$kcal/mol$]	$\Delta\Delta G$ Kartograf [$kcal/mol$]	$\Delta\Delta G$ Lomap2D [$kcal/mol$]	$\Delta\Delta G$ Lomap3D [$kcal/mol$]
1	9	−2.15	–	-3.5 ± 0.04	–
1	14	0.62	-1.2 ± 0.1	–	-1.1 ± 0.19
2	3	−0.69	–	0.37 ± 0.1	0.35 ± 0.02
2	14	−0.96	-2.4 ± 0.18	–	–
3	9	−3.04	–	-3.7 ± 0.11	–
3	11	−1.35	–	–	-3.9 ± 0.14
3	15	2.05	1.6 ± 0.05	–	–
5	10	6.71	8.4 ± 0.07	8.4 ± 0.1	8.4 ± 0.1
6	9	0.15	–	-0.04 ± 0.03	–
6	14	2.92	2.0 ± 0.04	–	2.1 ± 0.04
8	9	−6.19	–	-4.1 ± 0.05	–
8	14	−3.42	-2.1 ± 0.08	–	-2.1 ± 0.14
9	12	6.57	6.8 ± 0.04	–	–
9	14	2.77	–	2.0 ± 0.03	2.1 ± 0.11
9	16	0.47	–	-0.33 ± 0.08	–
10	15	0.43	1.4 ± 0.1	1.4 ± 0.16	1.5 ± 0.08
11	14	1.08	–	1.7 ± 0.09	–
11	16	−1.22	-0.75 ± 0.12	–	-0.67 ± 0.07
12	15	−1.48	-1.1 ± 0.04	-1.3 ± 0.08	-1.1 ± 0.05
13	14	0.22	0.09 ± 0.05	0.04 ± 0.12	0.11 ± 0.01
14	15	2.32	3.6 ± 0.02	3.6 ± 0.04	3.6 ± 0.06
15	16	−4.62	-6.2 ± 0.03	–	-6.1 ± 0.04

Table S2: RHFE $\Delta\Delta G$ - MST Network - The table provides the results of the free energy calculations with the three mapping approaches.

3 Additions to RBFE Simulations

3.1 TYK2

3.1.1 Radial Networks

ligA	ligB	experiment [$kcal/mol$]	$\Delta\Delta G$ Kartograf [$kcal/mol$]	$\Delta\Delta G$ Lomap2D [$kcal/mol$]	$\Delta\Delta G$ Lomap3D [$kcal/mol$]
ejm42	ejm31	0.25	0.08 ± 0.03	0.42 ± 0.07	0.31 ± 0.13
ejm43	ejm31	−1.29	0.75 ± 0.13	0.91 ± 0.24	0.98 ± 0.19
ejm44	ejm31	−2.14	2.2 ± 0.18	2.2 ± 0.16	2.0 ± 0.57
ejm45	ejm31	0.02	-0.23 ± 0.26	0.09 ± 0.26	0.41 ± 0.11
ejm46	ejm31	1.79	-1.6 ± 0.15	-1.6 ± 0.11	-1.4 ± 0.07
ejm47	ejm31	0.16	–	0.4 ± 0.28	0.15 ± 0.08
ejm48	ejm31	−0.54	0.95 ± 0.17	0.63 ± 0.15	0.69 ± 0.34
ejm49	ejm31	−1.81	0.27 ± 0.08	0.31 ± 0.2	0.4 ± 0.37
ejm50	ejm31	−0.57	0.39 ± 0.05	0.86 ± 0.1	0.91 ± 0.06
ejm54	ejm31	1.0	-0.6 ± 0.29	-0.81 ± 0.32	-0.96 ± 0.27
ejm55	ejm31	−0.34	-0.73 ± 0.23	-0.99 ± 0.22	-1.2 ± 0.18
jmc23	ejm31	2.18	-1.6 ± 0.07	–	–
jmc27	ejm31	1.75	-2.1 ± 0.13	-1.7 ± 0.12	-1.5 ± 0.24
jmc28	ejm31	1.45	-0.37 ± 0.13	-0.42 ± 0.28	-0.3 ± 0.16
jmc30	ejm31	1.41	-1.9 ± 0.15	-1.7 ± 0.19	-2.0 ± 0.25

Table S3: TYK2 $\Delta\Delta G$ - Radial Network - The table provides the results of the free energy calculations with the three different mapping approaches.

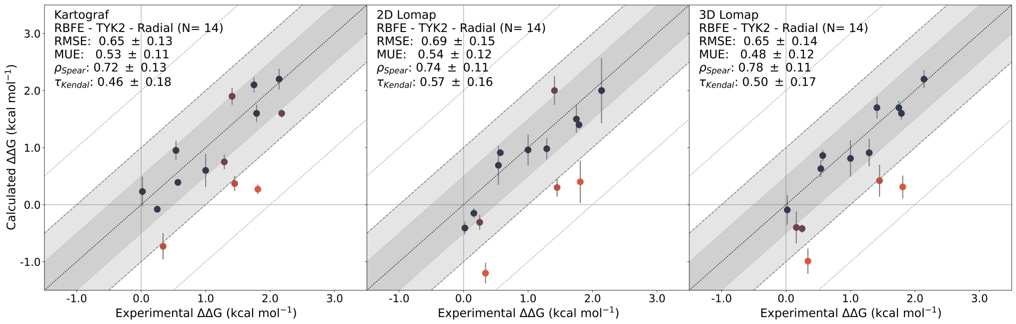

Figure S13: Relative binding free energies for the radial TYK2 dataset plotted against the experimental results.

3.1.2 MST Networks

ligA	ligB	experiment $[kcal/mol]$	$\Delta\Delta G$ Kartograf $[kcal/mol]$	$\Delta\Delta G$ Lomap2D $[kcal/mol]$	$\Delta\Delta G$ Lomap3D $[kcal/mol]$
ejm42	ejm31	0.25	0.1 ± 0.1	–	0.27 ± 0.04
ejm43	ejm42	-1.54	0.65 ± 0.13	0.8 ± 0.06	1.6 ± 0.09
ejm44	ejm43	-0.85	–	2.2 ± 0.09	1.9 ± 0.1
ejm45	ejm31	0.02	-0.04 ± 0.17	0.29 ± 0.21	–
ejm46	ejm31	1.79	-1.7 ± 0.15	-1.6 ± 0.09	-1.8 ± 0.2
ejm47	ejm31	0.16	0.15 ± 0.34	–	0.36 ± 0.26
ejm48	ejm31	-0.54	0.79 ± 0.08	0.68 ± 0.15	0.78 ± 0.09
ejm49	ejm31	-1.81	-0.0 ± 0.19	0.25 ± 0.36	0.1 ± 0.13
ejm50	ejm31	-0.57	–	0.74 ± 0.04	–
ejm50	ejm42	-0.82	0.51 ± 0.1	0.67 ± 0.12	–
ejm54	ejm31	1.0	–	–	-1.4 ± 0.17
ejm55	ejm31	-0.34	–	–	-0.74 ± 0.13
ejm55	ejm42	-0.59	-0.82 ± 0.2	-0.81 ± 0.1	–
ejm55	ejm45	-0.36	–	–	-0.97 ± 0.13
ejm55	ejm54	-1.34	-0.47 ± 0.16	-0.48 ± 0.06	–
jmc23	ejm46	0.39	–	–	-0.07 ± 0.06
jmc27	ejm46	-0.04	-0.27 ± 0.06	-0.35 ± 0.04	-0.16 ± 0.07
jmc28	ejm46	-0.34	–	–	1.1 ± 0.16
jmc28	jmc23	-0.73	1.3 ± 0.1	1.1 ± 0.16	–
jmc28	jmc27	-0.3	1.3 ± 0.13	1.4 ± 0.06	–
jmc30	ejm46	-0.38	-0.48 ± 0.02	-0.49 ± 0.12	-0.38 ± 0.08

Table S4: TYK2 $\Delta\Delta G$ - MST Network - The table provides the results of the free energy calculations with the three different mapping approaches.

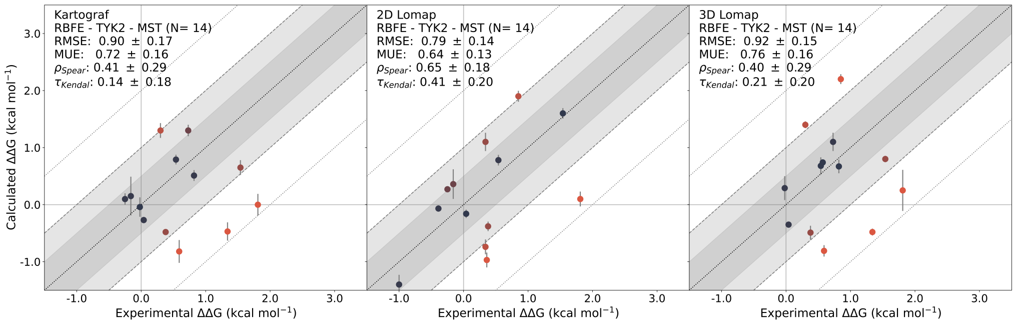

Figure S14: Relative binding free energies for the MST TYK2 dataset plotted against the experimental results.

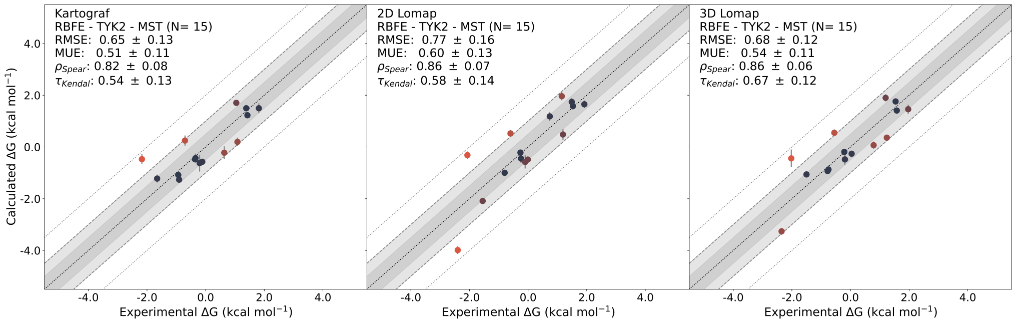

Figure S15: MLE derived absolute binding free energies for the MST TYK2 dataset plotted against the experimental results.

3.2 HIF2A

3.2.1 Radial Networks

ligA	ligB	experiment [$kcal/mol$]	$\Delta\Delta G$ Kartograf [$kcal/mol$]	$\Delta\Delta G$ Lomap2D [$kcal/mol$]	$\Delta\Delta G$ Lomap3D [$kcal/mol$]
1	163	0.31	-1.3 ± 0.14	-1.1 ± 0.22	-1.3 ± 0.6
124	163	-0.89	-0.23 ± 0.4	0.74 ± 0.24	-0.61 ± 1.3
15	163	0.04	-2.1 ± 0.21	-2.3 ± 0.21	-2.2 ± 0.09
155	163	-0.51	-1.0 ± 0.04	-1.3 ± 0.11	-1.2 ± 0.19
156	163	-1.27	-0.67 ± 0.11	-0.42 ± 0.31	-0.34 ± 0.26
165	163	-1.77	1.9 ± 1.2	1.3 ± 0.77	2.0 ± 0.22
167	163	-0.34	-0.88 ± 0.18	-0.84 ± 0.28	-0.87 ± 0.15
206	163	-0.89	2.3 ± 0.19	2.4 ± 0.04	2.3 ± 0.11
215	163	-0.41	-0.92 ± 0.09	-0.94 ± 0.03	-0.75 ± 0.14
224	163	-1.35	-1.0 ± 0.84	-0.61 ± 0.47	-1.3 ± 0.64
227	163	-0.25	-3.5 ± 0.19	-3.3 ± 0.36	-4.0 ± 0.22
23	163	-3.94	1.5 ± 0.14	1.1 ± 0.3	0.58 ± 0.3
231	163	-0.99	0.11 ± 0.05	0.2 ± 0.04	-0.42 ± 0.07
234	163	-0.66	0.24 ± 0.06	$-$	$-$
235	163	-0.89	-1.7 ± 0.28	-1.9 ± 0.05	-1.7 ± 0.17
25	163	-0.73	-0.59 ± 0.36	-3.2 ± 0.93	-3.5 ± 0.43
251	163	-0.77	3.4 ± 0.23	3.1 ± 1.2	3.0 ± 0.52
252	163	-1.3	2.9 ± 0.58	2.9 ± 1.3	1.7 ± 0.75
254	163	-1.46	2.6 ± 0.36	2.3 ± 0.47	2.4 ± 0.56
256	163	-0.34	2.4 ± 0.49	3.1 ± 0.52	-0.04 ± 1.3
289	163	-0.1	0.14 ± 0.13	0.05 ± 0.19	-0.98 ± 0.2
290	163	-0.44	-3.1 ± 0.73	-2.9 ± 0.93	-3.0 ± 1.1
30	163	-0.44	-1.7 ± 0.19	-1.5 ± 0.23	-2.0 ± 0.33
31	163	-2.17	1.2 ± 0.44	0.8 ± 0.25	0.93 ± 0.17
336	163	-1.19	1.8 ± 0.17	2.1 ± 0.89	1.3 ± 0.69
338	163	-0.25	$-$	0.9 ± 0.3	1.0 ± 0.81
35	163	-3.86	-0.21 ± 1.1	-0.92 ± 0.45	-0.83 ± 0.52
41	163	-1.22	-1.3 ± 0.28	-0.41 ± 0.62	-1.4 ± 0.26
43	163	-2.31	0.66 ± 0.31	0.67 ± 0.16	-0.15 ± 0.56
50	163	-1.31	3.6 ± 0.23	0.7 ± 0.11	0.44 ± 0.2
54	163	-1.7	1.4 ± 0.25	1.2 ± 1.0	1.5 ± 0.55
57	163	-0.34	-3.0 ± 0.21	-2.5 ± 0.29	-2.5 ± 0.25
61	163	-0.9	0.56 ± 0.36	0.51 ± 0.16	0.64 ± 0.14
67	163	-0.84	-1.0 ± 1.0	-1.4 ± 0.29	-1.3 ± 0.6
84	163	-3.43	3.0 ± 0.28	2.6 ± 1.6	1.5 ± 1.3

Table S5: Hif2A $\Delta\Delta G$ - Radial Network - The table provides the results of the free energy calculations with the three different mapping approaches.

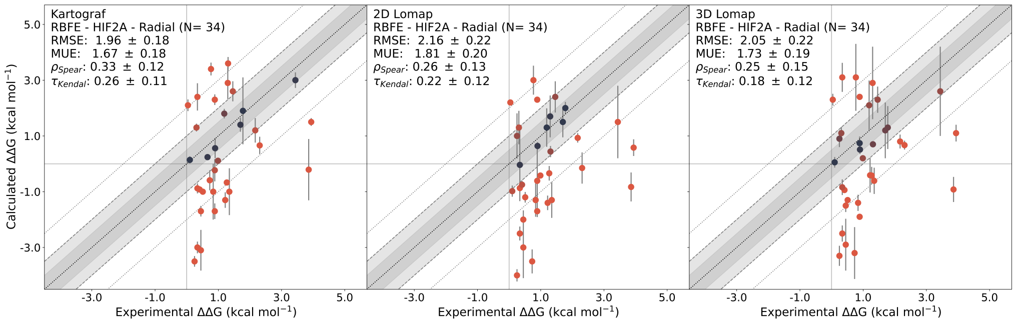

Figure S16: Relative binding free energies for the Radial HIF2A dataset plotted against the experimental results.

3.2.2 MST Networks

ligA	ligB	experiment [$kcal/mol$]	$\Delta\Delta G$ Kartograf [$kcal/mol$]	$\Delta\Delta G$ Lomap2D [$kcal/mol$]	$\Delta\Delta G$ Lomap3D [$kcal/mol$]
1	155	0.82	0.13 ± 0.2	-0.02 ± 0.07	-0.08 ± 0.15
156	155	-0.77	–	0.73 ± 0.13	0.75 ± 0.08
163	15	-0.04	–	–	2.3 ± 0.13
165	163	-1.77	1.9 ± 0.53	2.8 ± 0.6	2.3 ± 0.21
206	163	-0.89	–	–	2.3 ± 0.07
215	15	-0.45	1.1 ± 0.13	1.2 ± 0.1	–
215	163	-0.41	-0.79 ± 0.08	-0.84 ± 0.06	-0.97 ± 0.21
215	167	-0.07	-0.19 ± 0.11	-0.28 ± 0.13	–
215	206	0.48	-3.1 ± 0.08	-3.1 ± 0.1	–
227	15	-0.29	–	–	-1.4 ± 0.11
23	1	-4.26	2.5 ± 0.17	–	2.6 ± 0.17
231	163	-0.99	0.14 ± 0.09	0.11 ± 0.02	-0.63 ± 0.06
234	163	-0.66	0.2 ± 0.06	0.24 ± 0.07	0.22 ± 0.05
234	167	-0.33	–	–	1.1 ± 0.11
235	224	0.46	-1.1 ± 0.47	-1.2 ± 0.62	-1.5 ± 0.47
25	15	-0.76	–	-0.23 ± 0.55	1.6 ± 0.12
25	235	0.16	1.3 ± 0.13	–	2.3 ± 0.21
252	163	-1.3	2.9 ± 0.91	2.2 ± 0.24	–
254	206	-0.57	1.9 ± 0.38	1.5 ± 0.3	–
254	251	-0.69	-0.8 ± 0.14	-0.91 ± 0.06	-0.74 ± 0.22
254	252	-0.16	–	–	0.08 ± 0.49
256	251	0.43	1.3 ± 0.74	-1.2 ± 0.19	-2.0 ± 0.3
289	231	0.89	–	–	-0.82 ± 0.14
290	15	-0.48	-0.31 ± 1.0	-1.4 ± 0.17	–
290	165	1.33	–	–	-4.9 ± 0.05
290	227	-0.19	-0.02 ± 0.98	0.21 ± 0.58	–
290	235	0.44	-0.59 ± 1.3	-1.0 ± 0.42	–
30	155	0.06	–	-0.11 ± 0.24	-0.44 ± 0.24
31	30	-1.72	3.1 ± 0.09	3.0 ± 0.24	2.9 ± 0.17
336	289	-1.09	1.4 ± 0.29	1.4 ± 0.19	–
338	252	1.05	-1.0 ± 0.24	-1.2 ± 0.06	-1.3 ± 0.21
338	336	0.93	-0.57 ± 0.11	-0.47 ± 0.05	-0.59 ± 0.04
41	1	-1.54	0.4 ± 0.23	–	–
41	35	2.63	-0.12 ± 0.26	-0.32 ± 0.19	0.13 ± 0.12
43	31	-0.14	-1.0 ± 0.36	-1.0 ± 0.28	-1.1 ± 0.31
50	35	2.54	3.2 ± 0.65	3.8 ± 0.29	3.6 ± 0.44
54	124	-0.81	2.0 ± 0.74	2.4 ± 0.32	2.4 ± 0.05
54	41	-0.48	2.3 ± 0.39	2.0 ± 0.33	–
54	50	-0.39	–	–	-1.7 ± 0.2
57	155	0.17	-1.8 ± 0.11	-1.7 ± 0.18	-2.3 ± 0.08
57	156	0.94	-2.4 ± 0.14	–	–
57	227	-0.08	–	1.0 ± 0.05	1.6 ± 0.14
61	1	-1.22	1.8 ± 0.13	1.6 ± 0.15	2.0 ± 0.05
67	163	-0.84	-1.4 ± 0.66	-1.8 ± 0.34	-2.0 ± 0.29
84	1	-3.75	3.7 ± 0.3	4.8 ± 0.22	–
84	23	0.51	–	–	3.3 ± 0.3

Table S6: Hif2A $\Delta\Delta G$ - MST Network - The table provides the results of the free energy calculations with the three different mapping approaches.

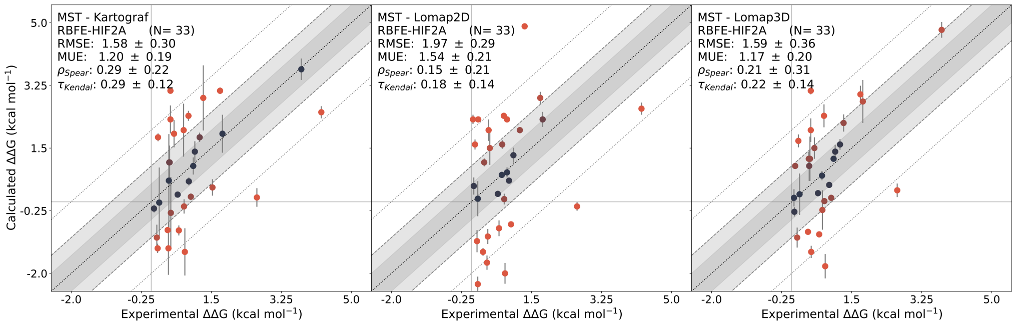

Figure S17: Relative binding free energies for the MST HIF2A dataset plotted against the experimental results.

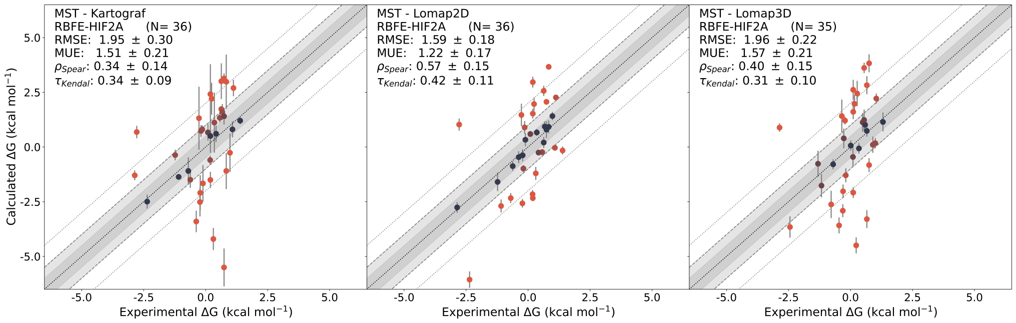

Figure S18: MLE derived absolute binding free energies for the MST HIF2A dataset plotted against the experimental results.
